# Building Goal-Directed Cognitive Graphs

**DOI:** 10.64898/2026.02.18.706628

**Published:** 2026-02-19

**Authors:** Adithya Gungi, Pradyumna Sepúlveda Delgado, Ines F. Aitsahalia, Marta Blanco-Pozo, Kiyohito Iigaya

**Affiliations:** 1 Department of Physics, Columbia University, New York, NY; 2 Department of Psychiatry, Columbia University Irving Medical Center, New York, NY; 3 The Italian Academy for Advanced Studies, Columbia University, New York, NY; 4 Center for Theoretical Neuroscience and Zuckerman Institute, Columbia University, New York, NY; 5 2CNC Program, Stanford University, Stanford, CA; 6 James H. Clark Center for Biomedical Engineering & Sciences, Stanford University, Stanford, CA; 7 New York State Psychiatric Institute, New York, NY; 8 Data Science Institute, Columbia University, New York, NY

## Abstract

Flexible, goal-directed behavior depends on learning predictive relationships, yet how reward shapes learned transition structure remains incompletely understood. Here we introduce the Sparse Cognitive Graph, a reinforcement-learning framework in which a continuously updated transition representation is sparsified into a directed graph that governs valuation and choice. Applied to human behavioral and mouse optogenetic datasets, the framework accounts for learning and decision-making across tasks and generates experimentally testable predictions. Differences in inferred graph topology give rise to distinct behavioral regimes despite unimodally distributed parameters, and dynamically evolving graphs reproduce canonical two-step task signatures within a unified framework. In mice, reward selectively enhanced learning of transitions preceding desirable outcomes, and dopamine stimulation produced behavioral effects consistent with enhanced transition learning. The model further predicts low-dimensional population signatures localized to graph entry and goal states. Together, these findings provide a mechanistic account of how reward-dependent learning biases internal relational structure to support flexible, goal-directed behavior.

## Introduction

A hallmark of biological intelligence is the ability to extract latent structure from experience and use that structure to guide goal-directed behavior. Increasing evidence suggests that such latent structure can be formalized as graph-like cognitive models (*cognitive graphs*^1–9^), in which latent states are linked through directional relationships that specify routes to desired outcomes.

Neural correlates of graph-like representations have been reported across multiple brain regions. Prefrontal neurons exhibit sequential and graph-like activity patterns associated with reward-related behavior.^10–17^ In parallel, hippocampal–entorhinal circuits support predictive cognitive maps across both spatial and abstract domains,^18–22^ and hippocampal input contributes to the development of representations in the prefrontal areas.^23–25^ Prefrontal regions also express low-dimensional relational and grid-like codes.^26–31^

Despite this growing body of neural evidence, a central computational question remains unresolved: how does compact, goal-directed relational structure emerge from predictive experience, and how is it reorganized as learning unfolds through interaction with reward? Computational frameworks such as the successor representation (SR) provide a principled account of predictive transition learning and have been closely linked to hippocampal–entorhinal coding.^3,32^ In these models, behavior is typically derived directly from a dense predictive map of state relationships. These approaches therefore do not explain how dense predictive representations give rise to compact, goal-directed graph structure that governs choice.

From a computational perspective, this distinction becomes increasingly relevant in large environments, where decisions directly based on dense predictive representations can be computationally costly^33^ and unnecessary.^34–36^ Consistent with this view, behavioral evidence suggests that people use various approximations in planning: pruning irrelevant transitions,^37^ restricting the depth of planning,^38^ or treating highly probable transitions as effectively deterministic.^39–41^ Even well-studied learning behavior, such as the canonical two-step behavior that is often interpreted as reflecting a mixture of model-based and model-free control,^42^ can also arise from heuristic or imperfect structural inference^43,44^ constrained by working memory capacity.^45^

Reward in the environment, and dopamine in the brain, are strong candidates for shaping internal structure during learning. Reward modulates hippocampal and prefrontal plasticity^46,47^ and can drive the acquisition of causal transitional structure,^48^ consistent with recent evidence that hippocampal representations become selectively biased toward states that predict future reward.^22^ Dopamine signals convey not only reward prediction errors,^49–51^ but also unexpected transitions,^43,52–61^ latent boundaries,^62–65^ hidden states,^66,67^ and policy shifts mediated by increased learning rates.^68,69^ Reward and dopamine can further bias cortical and hippocampal maps through several mechanisms: prioritized replay,^70–72^ goal-directed changes in place and grid fields,^73–75^ and relational remapping following changes in task contingencies.^76–81^ However, how reward and dopaminergic signals shape compact, goal-directed graph structure during learning remains unclear.

Here we introduce the *Sparse Cognitive Graph* (SCG), a reinforcement-learning framework that formalizes how compact graph-like representations can emerge from predictive experience. In SCG, agents continuously update a dense predictive representation that stores graded relationships among states. In parallel, a sparse directed graph is constructed by nonlinearly thresholding this representation. Crucially, valuation and choice are computed from the sparse graph rather than directly from the dense representation, so that predictive knowledge is maintained in full while only a subset of transitions becomes behaviorally operative. The SCG captures multimodal individual differences in human transition and reward revaluation, reproduces two-step task behavior without assuming fixed mixtures of model-based and model-free strategies, and provides a computational account of how reward and dopamine can bias the selective expression of relational structure. The framework further predicts low-dimensional start- and goal-related population signatures that arise from the topology of the learned graph and, under conditions of spatial or temporal translational invariance, can yield grid-like structure. Together, this work provides a computational account of how predictive learning gives rise to compact cognitive graphs that support flexible, goal-directed behavior.

## Results

### Building sparse cognitive graphs through reinforcement learning

**Figure 1:**
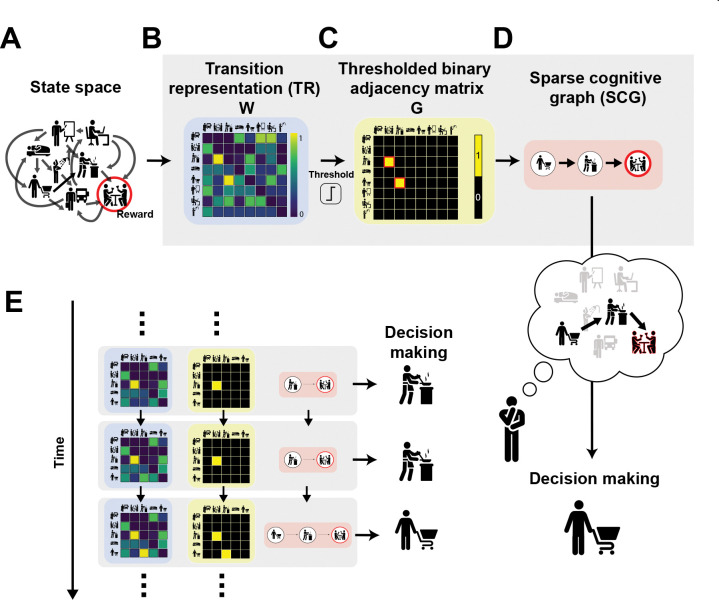
: Sparse Cognitive Graph (SCG): a reinforcement-learning model for graph construction. (**A**). An agent experiences sequential transitions between latent states. (**B**). From experience, the agent incrementally learns a transition representation W capturing discounted predictions of successor states. (**C**). After each update, W is thresholded to selectively retain sufficiently predictive transitions, producing a sparse directed adjacency matrix G. (**D**). This adjacency matrix G defines the Sparse Cognitive Graph, which serves as a compact representation used for valuation and choice. (**E**). Throughout learning, the dense transition representation W and the sparse graph G are updated in parallel. W continuously integrates new experience, while G captures a compact, goal-directed model that directly shapes value computation and decision making.

Here we formalize the Sparse Cognitive Graph (SCG), a reinforcement-learning framework that constructs a directed internal model from experience. As an agent interacts with an environment composed of directed state transitions (Fig. 1A), it incrementally learns a transition-anchored predictive representation W (Fig. 1B), which we term the *transition representation* (TR). The TR captures how each state predicts its successors under temporal discounting, preserving core properties of successor representation (SR) learning.^3,82^

We use the TR rather than the standard SR because our goal is to construct an explicit directed graph of experienced transitions. Whereas the SR encodes discounted future state occupancy, the TR is anchored to experienced one-step transitions and therefore provides a more direct substrate for graph construction. This distinction is clearest in the limit of zero discounting: the TR converges to the empirical one-step transition matrix, whereas the SR converges to the identity matrix (see Methods). As a result, the TR naturally supports the extraction of directed relational structure from ongoing experience.

After each experienced state transition, the updated TR (W) is used to construct a sparse binary adjacency matrix G via a simple thresholding operation (Fig. 1C). Elements of W that exceed a threshold ζ are retained as 1 (edge) in G, whereas all others are discarded to 0 in G. In graph-theoretic terms, G is a binary adjacency matrix that defines a directed graph whose nodes correspond to states and whose edges mark reliable transitions between them (Fig. 1D). We refer to this thresholded structure as the Sparse Cognitive Graph (SCG). In this manuscript we analyze the simplest instantiation of this process, using a fixed threshold applied uniformly across transitions and trials.

This sparsification step selectively retains transitions that have accumulated sufficient predictive support, yielding a compact internal structure that can support efficient planning and is consistent with known limits on working memory.^83^ Importantly, thresholding should be understood as a functional nonlinearity rather than a literal hard cutoff, and could potentially arise from multiple biological mechanisms operating online or offline, including synaptic consolidation, inhibitory competition, or replay-based pruning. Here we adopt a minimal algorithmic form that remains agnostic about neural implementation.

The model maintains both the dense transition representation W and the sparse cognitive graph G, which evolve jointly throughout learning (Fig. 1E). Each experienced transition triggers (i) a temporal-difference update to W and (ii) an online thresholding step that updates G. This co-learning procedure preserves long-range predictive information in W while selectively consolidating reliable transitions into an interpretable directed structure G. Because our focus here is on how inferred graph structure guides behavior, we assume that valuation and action selection operate directly on the sparse graph G, rather than the underlying dense predictive representation W. This contrasts with standard SR-based approaches, in which behavior is typically derived directly from dense predictive representations.^82^

Motivated by extensive behavioral and neural evidence that reward modulates learning rates,^48,66,84–88^ we allow the transition learning rate for updating W to depend on whether the observed transition was rewarded $\alpha_{\to R}$ or unrewarded $\alpha_{\to NoR}$. Importantly, we do not impose any a priori ordering or constraint on these learning rates; instead, we test for such asymmetries empirically.^66^

A computational motivation for allowing this flexibility is that, if reward-driven updates are larger $\alpha_{\to R}>\alpha_{\to NoR}$, transitions followed by reward strengthen more rapidly in W, and therefore are more likely to exceed threshold and be retained in the graph G relative to transitions leading to no reward. Through this mechanism, reward can bias graph formation toward goal-relevant pathways, not by explicitly encoding goals, but by accelerating the consolidation of transitions that reliably predict desirable outcomes.

### Sparse cognitive graphs explain multimodal individual differences in human structure learning

To test whether the SCG can account for human structural learning tied to reinforcement, we applied the model to reward and transition revaluation task data originally reported by Momennejad and colleagues.^89^ These tasks are intentionally challenging and do not assume that participants recover the full task structure; instead, they are designed to reveal how individuals simplify and abstract relational structure under limited experience. Participants exhibit discrete patterns of responses, an empirical feature that so far lacks a mechanistic explanation. We therefore asked whether the SCG can account for this behavioral diversity by modeling how simplified internal structure is inferred and updated.

In both tasks, participants first learned two three-step sequences leading to different reward outcomes:

A→B→C→$10,D→E→F→$1.

**Figure 2:**
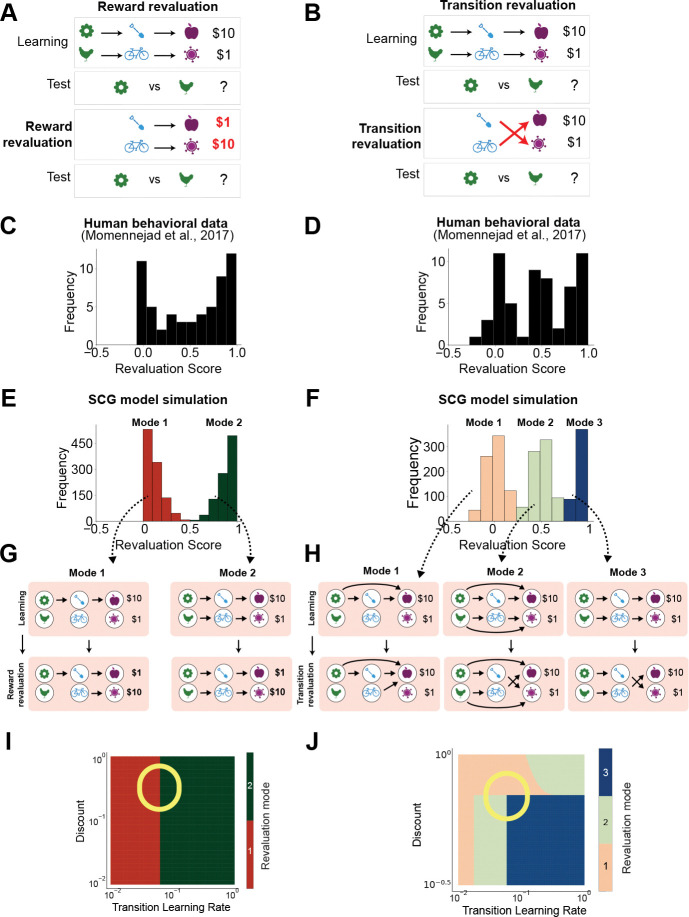
: Sparse cognitive graphs explain multimodal human structure learning.^89^ (**A**). Reward revaluation task. Participants learned two independent three-step sequences leading to a large ($10) or baseline ($1) reward. During revaluation, terminal reward contingencies were swapped without re-exposure to the starting states. Participants reported their relative preference between the two starting states before and after revaluation learning. (**B**). Transition revaluation task. After initial learning, the transition between the second and third states was swapped, while reward associations with the terminal states remained unchanged. (**C**). Behavioral data from the reward revaluation task.^89^ Participants’ revaluation scores exhibited a clear bimodal distribution. (**D**). Behavioral data from the transition revaluation task.^89^ Revaluation scores exhibited a trimodal distribution. (**E**). SCG model simulations for reward revaluation. The model reproduced the bimodal distribution observed in data, despite being simulated with a unimodal parameter distribution. (**F**). SCG model simulations for transition revaluation. The model reproduced the trimodal distribution observed in data, despite being simulated with a unimodal parameter distribution. (**G**). Graphs generated by the SCG in the reward revaluation task. The model produced two distinct graph structures corresponding to no change in preference or complete reversal. (**H**). Graphs generated by the SCG in the transition revaluation task. The model produced three distinct graph structures corresponding to no change in preference, complete reversal, or becoming indifferent. (**I**). The phase diagram of the SCG in the reward revaluation task revealed two distinct behavioral regimes. Discount factor γ and learning rate ($\alpha_{\to NoR}$) were systematically varied. This phase transition enables bimodal behavioral distributions to emerge from a unimodal parameter distribution. The circle marks the parameters used for the simulations. (**J**). Phase diagram of the SCG in the transition revaluation task reveals three distinct behavioral regimes.

In the reward revaluation task (Fig. 2A), during the next revaluation phase the reward magnitudes associated with the terminal states were swapped,

B→C→$1,E→F→$10,

while the initial states A and D were not revisited. Participants therefore had to infer how the change in reward propagated backward through the previously learned transition structure.

In the transition revaluation task (Fig. 2B), during the revaluation phase reward values were held fixed but the intermediate transitions were altered,

B→F→$1,E→C→$10.

Again, the initial states were not re-experienced, requiring participants to update starting-state values through inferred changes in latent task structure rather than through direct reinforcement.

Behavioral data in these tasks exhibited clear multimodal structure, a striking feature that has not previously been examined mechanistically.^89^ Revaluation was quantified using a revaluation score,^89^ defined as the normalized change in relative preference between the two initial states A and D from the end of initial learning to the test phase after revaluation. By construction, a revaluation score of 0 indicates no change in preference, a score of 1 indicates complete reversal from A to D, and a score of 0.5 indicates indifference. In the reward revaluation task, revaluation scores were bimodally distributed, with one group showing no change in preference and another showing complete reversal (Fig. 2C).^89^ In the transition revaluation task, revaluation scores were trimodally distributed, corresponding to no change, full reversal, or indifference (Fig. 2D).^89^

These discrete behavioral regimes suggest that participants may infer qualitatively different internal representations of task structure despite experiencing the same objective task. To test this idea, we applied the SCG model to these tasks. In simulations, the SCG captures the observed multimodal behavioral patterns as a direct consequence of distinct inferred graph configurations (Fig. 2E–F). Notably, model agents were drawn with a unimodal parameter distribution and trained on identical task experiences, yet diverged into distinct graph structures (Fig. 2G–H, Fig. S1), yielding multimodal behavioral distributions. These distinct graph structures produced qualitatively different valuations of the starting states A and D, corresponding closely to the observed behavioral regimes.

Phase diagram analyses revealed how these multimodal patterns arise (Fig. 2I–J). Varying model parameters exposed discrete regimes of inferred graph structure, with two regimes in reward revaluation and three regimes in transition revaluation. Because graph construction involves a nonlinear thresholding operation, small differences in parameters near the boundaries between regimes produced abrupt changes in inferred graph structure and choice. This nonlinearity allows multimodal behavioral distributions to emerge even when underlying parameters are smoothly and unimodally distributed. In these simulations, transition learning rates were allowed to differ between transitions yielding $10 and $1 outcomes, with the smaller outcome ($1) treated as a baseline so that learning-rate modulation reflects the presence $\alpha_{\to R}$ versus absence $\alpha_{\to NoR}$ of additional reinforcement ($9) rather than reward magnitude.

By contrast, standard computational models, including the classical successor representation, model-free temporal difference learning, fully model-based reinforcement learning, and standard mixtures of model-based and model-free controllers, at least in their commonly used forms, did not reproduce the observed multimodal distributions when parameters were drawn from unimodal distributions, instead producing unimodal or smoothly varying behavior (Fig. S2). Notably, models that rely on dense predictive representations without nonlinear sparsification, such as the SR, failed to produce these discrete behavioral regimes, highlighting the importance of graph thresholding for capturing the observed structure.

Together, these results suggest that nonlinear graph construction provides a potential mechanistic account of multimodal individual differences observed in these human structure learning tasks.

### Sparse cognitive graphs reproduce human two-step task behavior

**Figure 3:**
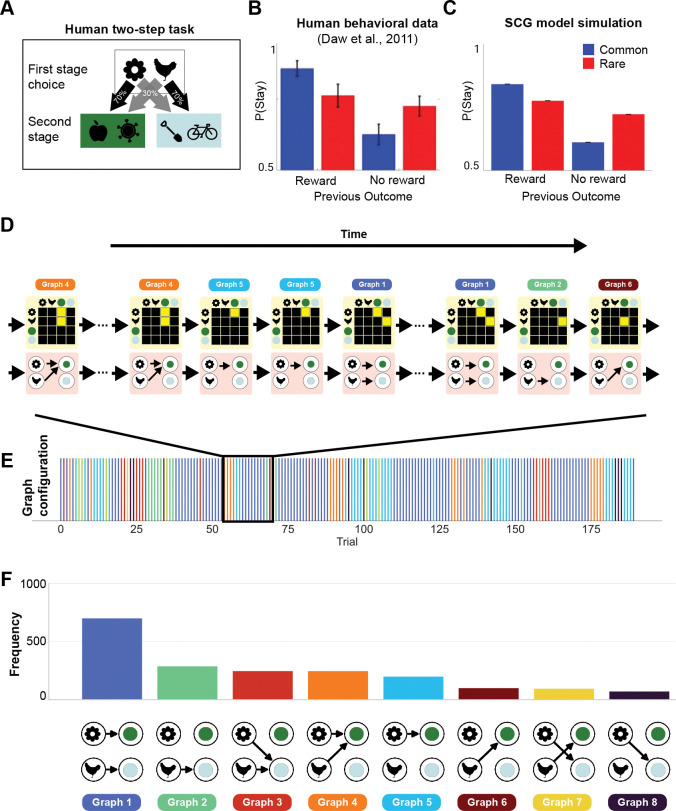
: Sparse cognitive graphs reproduce canonical human two-step task behavior. (**A**). Two-step task. On each trial, participants choose between two first-stage options that make common (70%) and rare (30%) transitions to second-stage options. The second stage served to reveal probabilistic rewards, with reward probabilities assigned to each option drifting slowly over time according to independent random walks. (**B**). Human behavior. Stay probability depends jointly on reward outcome and transition type, a canonical signature often interpreted as reflecting a mixture of model-based and model-free control. Reproduced from.^42^Error bars indicate s.e.m. across participants. (**C**). SCG model simulations reproduced this behavioral pattern. Error bars indicate s.e.m. across task simulations. (**D**). During simulation, the SCG model continuously reconfigured graph structure across trials according to recent experience. (**E**). Temporal evolution of graph configurations across trials, with colors indicating distinct graph structures expressed at different points in learning. (**F**). Distribution of graph frequencies across trials. The most frequent graph (Graph 1) retained only common transitions.

We next asked whether the SCG could account for behavior in the widely studied two-step task.^42^ In this task, on each trial (Fig. 3A), participants choose between two first-stage options, each of which leads probabilistically to one of two second-stage states, with one transition common (70%) and the other rare (30%). Participants then make a second-stage choice, with reward probabilities that drift slowly over time according to random walks.

Behavior in this task is commonly interpreted as reflecting a mixture of model-based and model-free reinforcement learning.^42,45^ This interpretation is summarized by the canonical stay probability analysis (Fig. 3B), which quantifies the probability of repeating a first-stage choice as a function of the previous trial’s reward outcome and transition type. Model-free learning predicts choice repetition following reward regardless of transition, whereas model-based learning predicts an interaction between reward and transition to determine choice. Empirical data typically show signatures of both patterns, motivating mixture-based explanations. However, accumulating evidence indicates that similar behavioral signatures can arise from alternative computational mechanisms and depend sensitively on representational assumptions and task framing.^43–45,90^

We found that the SCG reproduces the hallmark stay probability interaction without requiring an explicit mixture of model-based and model-free controllers (Fig. 3C). Simulations used a representative parameterization chosen to illustrate this behavior (see Methods). In the SCG, behavior is governed by a dynamically evolving internal graph rather than a fixed transition model or fixed arbitration between controls. As agents accumulate experience, the learned graph structure reconfigures over trials (Fig. 3D–E), resulting in a distribution of internal graphs that are expressed across the session (Fig. 3F). This ongoing reconfiguration of sparse graph structure is sufficient, in principle, to generate the characteristic reward-by-transition interaction observed in human behavior (Fig. 3B,C).

While alternative explanations remain possible,^43,44,90^ these results suggest that canonical two-step behavioral patterns can potentially arise from dynamically inferred graph structure, without relying on an explicit mixture of model-based and model-free strategies.

### Reward and dopamine facilitate sparse cognitive graph learning in mice

**Figure 4:**
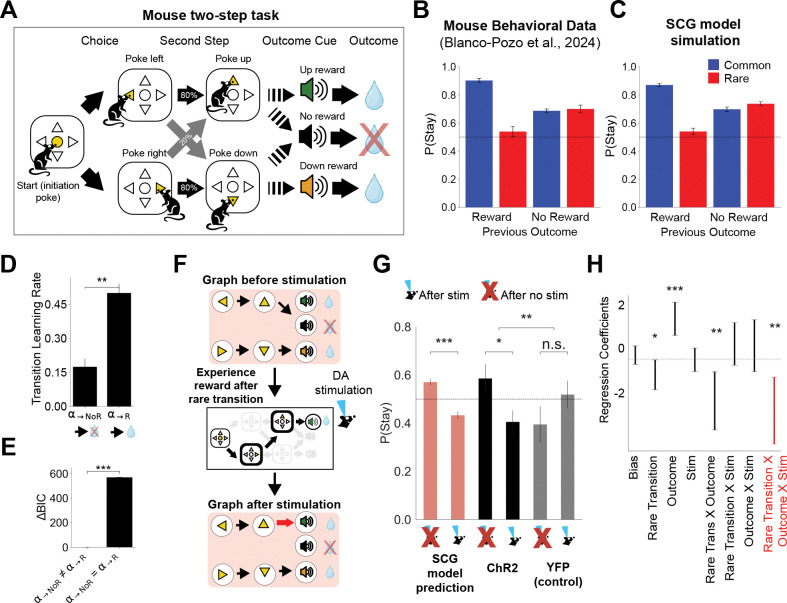
: Reward and dopamine facilitate sparse cognitive graph learning. Data originally reported in.^66^ (**A**). Mouse two-step task,^66^ which parallels the human paradigm. On each trial, mice chose between left and right options at the first stage, which led probabilistically to the presentation of an upper or lower stimulus at the second stage. Common (80%) and rare (20%) transitions linked first- and second-stage states. After the second stage, a state-specific reward cue (up-reward or down-reward) was presented on rewarded trials, whereas a state-unspecific cue was presented on unrewarded trials. (**B**). Behavioral data. Mouse choice patterns differed from those observed in humans and were not well explained by standard model-based or model-free reinforcement learning accounts, as previously reported.^66^ (**C**). SCG simulations reproduced the observed mouse behavioral pattern. Model parameters were fit to trial-by-trial choice data.^66^ Error bars indicate s.e.m. across subjects. (**D**). The transition learning rate following reward was significantly higher than the learning rate following no reward ($\alpha_{\to R}>\alpha_{\to NoR}$), consistent with reward selectively enhancing graph learning for transitions leading to rewarded states. Error bars indicate s.e.m. across subjects. (**E**). Formal model comparison using the integrated Bayesian Information Criterion (iBIC) favored the model with asymmetric transition learning ($\alpha_{\to R}\neq \alpha_{\to NoR}$) over the symmetric learning model ($\alpha_{\to R}=\alpha_{\to NoR}$). Smaller iBIC values indicate better model fit. Error bars indicate the cross-sample mean ± s.e.m. (4,000 bootstrap samples from the posterior distribution). (**F**). Illustrative example of how optogenetic stimulation of dopamine neurons can induce graph reconfiguration, leading to shifts in subsequent choice. Optogenetic stimulation at outcome was modeled as an increase in the transition learning rate, analogous to reward. (**G**). Model prediction and behavioral data. The model predicts reduced stay probability following optogenetic stimulation on rewarded rare-transition trials. Behavioral data from the Channelrhodopsin-2 (ChR2) group showed this effect (*p* < 0.05, permutation test), whereas the yellow fluorescent protein (YFP) control group showed no significant effect. The effect was significantly stronger in the ChR2 group than in the YFP control group (*p* < 0.01, permutation test). Error bars indicate s.e.m. across subjects. (**H**). Regression analyses predicting stay (positive) versus switch (negative) behavior further supported the model prediction that dopamine neuron stimulation enhances graph formation. The three-way interaction Rare Transition × Reward × Stimulation was significantly negative in the ChR2 group (*p* < 0.01), indicating increased switching following rewarded rare transitions under stimulation, and was absent in the control group (see Fig. S7). Error bars show ±2 SE (approx. 95 % CI).

To test the model more quantitatively and to assess whether the SCG generalizes across species, we next examined a recently published mouse two-step task dataset.^66^ In this task variant (Fig. 4A), mice first chose between two first-stage targets (left vs. right). Each choice probabilistically led to one of two second-stage ports (up vs. down), with common (80%) and rare (20%) transitions. At the second stage, a tone specific to each port indicated whether reward would be delivered, whereas white noise signaled the absence of reward. Reward probabilities depended on two block types: in balanced blocks both ports yielded equal reward likelihood, whereas in biased blocks one port was more rewarding. Block switches were unsignaled.

The original study reported three notable observations.^66^ First, unlike humans, mouse behavior substantially deviated from predictions of standard mixtures of model-based and model-free reinforcement learning (Fig. 4B), suggesting potentially task-specific hidden state inference.^66^ Second, the data suggested asymmetric learning from reward versus no-reward outcomes.^66^ Third, optogenetic stimulation of ventral tegmental area (VTA) dopamine neurons at outcome cues did not shift choice behavior in the manner predicted by standard RL models, although it reliably shortened inter-trial intervals.^66^ To account for these findings, the authors proposed a recurrent network model implementing hidden-state inference.^66^

We asked whether the SCG framework could account for the mice behavior in this task. We instantiated the SCG model with a state space aligned to the task structure and fit the model’s parameters to trial-by-trial choice data.^37,91^ The model reproduced the observed behavioral pattern (Fig. 4C). As in human two-step task, this was achieved through dynamic updates of sparse graph configurations across trials, yielding a distribution of inferred graph structures over trials (Fig. S3).

Importantly, fitted SCG model parameters indicated that transition learning rates in W were larger for rewarded than unrewarded transitions across animals (Fig. 4D; $\alpha_{\to R}>\alpha_{\to NoR}$; permutation test p<0.001). Formal model comparison using the integrated Bayesian Information Criterion (iBIC) with a Bayesian hierarchical framework further supported this result:^37,91^ the model with asymmetric learning rates $\alpha_{\to R}\neq \alpha_{\to NoR}$ outperformed an otherwise identical model with a single symmetric learning rate $\alpha_{\to R}=\alpha_{\to NoR}$ (Fig. 4E). While this is generally consistent with prior work showing enhanced learning after reward,^48,66,84–88^ within the SCG framework it has a specific structural interpretation: reward accelerates the accumulation of predictive support for recent transitions, thereby biasing graph formation toward pathways that precede reward. This provides a computational account for why rewarded trajectories may become preferentially expressed in inferred goal-directed cognitive graph structure.

By contrast, other standard computational models, including the classical SR, model-free temporal difference learning, and model-based reinforcement learning, were unable to reproduce the observed mouse behavioral patterns (Fig. S4), consistent with previous reports.^66^ Formal hierarchical model comparison further favored the SCG over these alternatives, including the best-performing model from the original analysis (a model-based agent with asymmetric reward learning rates^66^) (Fig. S5). Notably, the SR model, which relies on a dense predictive representation without explicit sparsification, did not account for the data under the parameter regimes examined, highlighting the role of graph construction and thresholding in accounting for behavior in this task.

Building on this framework, we next asked whether the SCG could account for the effects of optogenetic stimulation in the same dataset.^66^ The original analysis reported no reliable effect of dopamine neuron stimulation at outcome timing on subsequent choice, posing a challenge for interpretations in which dopamine acts solely as a value update signal in reinforcement learning. Within the SCG framework, however, reward has two distinct computational roles that could potentially be mediated by dopamine. The first is the conventional reward prediction error used to update the reward vector r. The second, highlighted by our model analyses (Fig. 4DE), is a modulation of transition learning rates, thereby accelerating graph formation.

Because the value-update interpretation was thoroughly evaluated in the original study,^66^ we focused here on this second, structural mechanism. Specifically, we asked whether modeling dopamine stimulation as an increase in transition learning rates would generate a distinct, testable behavioral signature. Accordingly, we implemented stimulation as a transient increase in transition learning rates within the model. Under this assumption, the SCG makes a testable behavioral prediction. A single rewarded rare transition is typically insufficient to modify the graph; however, when reward is paired with stimulation, the amplified update can promote the formation of a new reward-linked edge. This reconfiguration of graph structure predicts an increased tendency to switch on the subsequent trial following stimulation on rewarded rare transitions (Fig. 4FG), particularly under balanced reward-probability conditions where baseline stay probabilities are near chance levels.

We tested this prediction directly in the behavioral data. Consistent with the model’s prediction, mice exhibited significantly reduced stay probabilities following stimulation on rewarded rare-transition trials compared with no-stimulation trials and with control animals (Fig. 4G, Fig. S6; p<0.01, permutation test). Regression analyses also independently confirmed this effect (Fig. 4G), which was absent in control animals (Fig. S7).

Previous studies have suggested that the presence of a cognitive graph is associated with shorter reaction times in humans.^92^ Consistent with this idea, inter-trial intervals in mice were significantly shorter following stimulation at outcome compared with no-stimulation event,^66^ suggesting that stimulation may facilitate the formation or stabilization of graph structure.

Together, these results support a computational interpretation in which reward and dopamine stimulation facilitate graph edge formation. This mechanism complements, rather than replaces, established roles of reward and dopamine in reinforcement learning.

### Sparse cognitive graphs predict flag-like and grid-like low-dimensional population signatures

Beyond its behavioral consequences, we asked how the structure of internal models might give rise to low-dimensional population-level neural activity patterns. Previous studies have shown that low-dimensional spectral decompositions of the SR yield grid-like periodic structure in spatial environments.^3,93^ This phenomenon arises as a mathematical consequence of approximate translational invariance in predictive structure under 1D and 2D random walk navigation.^3,93^ Here, we asked what low-dimensional neural structure is predicted when predictive structure is sparse and directional, as in the SCG. The analyses below concern the spectral structure of the model’s adjacency matrix and generate predictions about potential population-level neural signatures.

**Figure 5:**
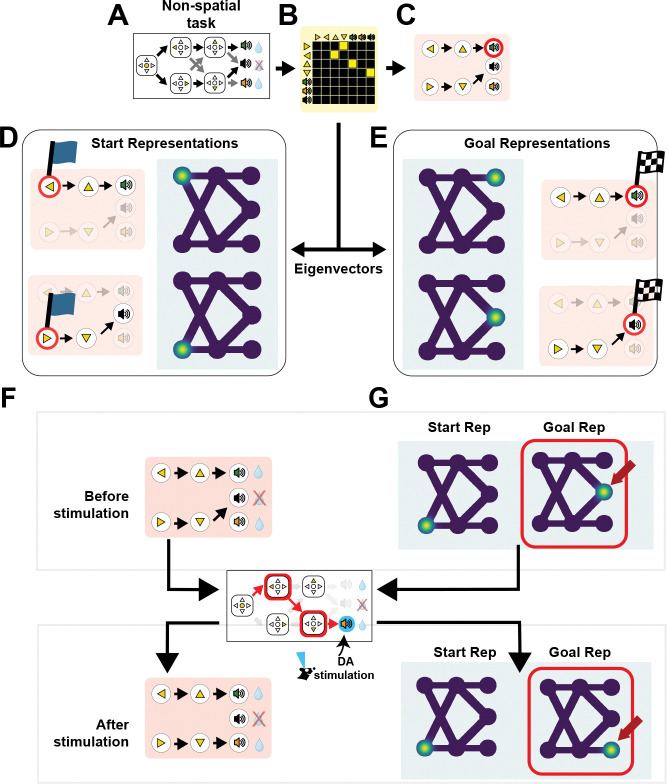
: Sparse cognitive graphs predict flag-like population signatures. (**A**). Simulated mouse two-step task.^66^ (**B**). Example sparse adjacency matrix G. (**C**). Directed graph derived from G. (**D**). Start-selective population signatures. Right eigenvectors of G yield activity modes localized to the starting states of each directed subgraph. (**E**). Goal-selective population signatures. Left eigenvectors of G yield activity patterns localized to the terminal states of each subgraph. Note that these signatures are expected to be expressed at the population level rather than by single neurons. (**F**). SCG prediction for dopamine-driven reorganization of cognitive graphs, low-dimensional neural modes. Pairing dopamine stimulation at outcome after a specific transition (e.g., a rare transition) selectively strengthens the corresponding edge, facilitating graph reorganization. (**G**). Changes in graph topology alter the eigenstructure of the SCG, shifting graph boundaries and reorganizing the expression of flag-like population signatures.

We applied the same standard spectral analyses^3^ to the SCG adjacency matrix G learned in the two-step task (Fig. 5A–C). This revealed a qualitatively distinct pattern from grid-like representations. The right eigenvectors of G were sharply localized at the starting states of directed subgraphs (Fig. 5D), whereas left eigenvectors peaked at corresponding terminal (goal) states (Fig. 5E). These complementary start- and goal-localized eigenmodes reflect how directed reachability in the SCG emphasizes entry and terminal states within learned subgraphs. We refer to these model-derived patterns as *flag-like* modes, to distinguish them from grid-like representations that tile state space. Importantly, the SCG does not assume specialized tuning at the level of single neurons; instead, these signatures arise at the population level as low-dimensional patterns of activity. While such structure could, in principle, be expressed at the level of individual neurons (potential “flag cells”), the model neither assumes nor requires single-neuron selectivity.

Whether low-dimensional structure is boundary-localized or periodic depends on the cyclicity of the underlying predictive structure. Directed, non-cyclic graphs emphasize sources and sinks, giving rise to boundary-localized flag-like signatures. This property is not unique to the SCG: even dense predictive operators, including the transition representation W or the SR, can exhibit eigenmodes localized at sources or sinks when translational invariance is broken by task structure or spatial geometry, such as environmental boundaries.^94^ By contrast, many behavioral tasks are intrinsically cyclic. Animals often experience repeating trial structure linking reward states, inter-trial intervals, and subsequent trial onset, and can exploit this cyclicity.^95^ In such regimes, dense predictive representations naturally support periodic low-dimensional structure, including grid-like patterns.

Within the SCG framework, whether flag-like or grid-like signatures dominate depends on the cyclicity of the thresholded graph G. When the graph remains non-cyclic, for instance, when connections through the inter-trial interval are weak or absent, sparsification emphasizes directed reachability to goals and gives rise to boundary-localized start- and goal-selective population signatures (Fig. 5DE). By contrast, after extensive training that induces a loop linking the reward state to subsequent trial onset, the learned graph acquires an effective translational symmetry imposed by the repetitive trial structure. In this regime, the SCG predicts grid-like periodic eigenstructure under conditions that approximate translational invariance, even in non-spatial tasks such as the two-step paradigm (Fig. S8A). Extending the model to a two-dimensional navigation environment yields a similar transition (Fig. S8B). When sparsification is sufficiently weak to preserve dense, approximately symmetric connectivity, the SCG reproduces grid-like structure analogous to that derived from the SR.^3^

Together, these results indicate that grid-like representations arise in regimes of dense, symmetric predictive structure, whereas sparse, directed cognitive graphs predict low-dimensional population signatures that are boundary- and goal-localized, reflecting the computational organization of goal-directed behavior.

### Prediction: dopamine-driven graph reorganization reshapes population signatures and choice

Building on these results, the SCG framework makes a concrete and testable prediction for how dopamine modulates internal models by facilitating the reshaping of cognitive graph structure. In particular, dopamine stimulation is predicted to alter the topology of learned graphs, thereby reorganizing low-dimensional population-level neural signatures and behavior. We illustrate this prediction in the context of the two-step task.

If a mouse experiences a rare transition, enters the Up state, and obtains reward (Fig. 5F), pairing this outcome with optogenetic dopamine stimulation is predicted to increase the effective learning rate for that transition. In the SCG, this selectively strengthens the edge along the experienced path and can introduce a new directed connection in the graph.

In the model, altering graph topology reorganizes the graph’s spectral structure, which in turn predicts shifts in start- and goal-selective population-level activity patterns (Fig. 5G). Because flag-like signatures reflect the directional geometry of the SCG, the model predicts that population-level activity patterns could provide a readout of how dopamine-dependent learning reshapes inferred graph structure. These predictions are directly testable using population-level neural recordings combined with targeted manipulation of dopamine signaling. Importantly, the model does not assume start- or goal-selective tuning at the single-neuron level; instead, changes in graph-dependent selectivity can emerge at the level of neural populations.

As a more speculative extension, the SCG framework suggests that dopamine stimulation could, in principle, promote the formation of cyclic graph structure. For example, pairing stimulation with states encountered during the inter-trial interval could promote the formation of loops in the graph. If such loops confer approximate translational invariance, the resulting population signatures are predicted to exhibit grid-like periodicity, even in two-step tasks that are not explicitly spatial (Fig. S8A).

## Discussion

Understanding how brains extract compact, goal-relevant structure from complex experience is a central challenge in cognitive neuroscience. While increasing evidence suggests that behavior can be guided by graph-like internal models, how such models are learned and shaped by reward remains unclear. Here, we introduce the Sparse Cognitive Graph (SCG), a reinforcement-learning framework in which a predictive transition representation is selectively consolidated into a sparse, directed graph that governs valuation and choice. Across the tasks studied here, the SCG accounts for multimodal patterns of human structure learning^89^ and canonical features of the two-step task in humans and mice.^42,66^ More broadly, the framework enables principled inference about internal models and generates testable predictions about population-level neural signatures and their reorganization. The central contribution of the SCG is not a specific update rule, but the principle that internal models emerge through nonlinear selection of predictive transitions, with reward biasing which connections become behaviorally expressed.

### Graph sparsification: behavioral expression and computational efficiency.

A key feature of the SCG is the shift in behavioral control from dense transition representations W to a thresholded sparse graph G. This nonlinear thresholding leads to abrupt changes in graph connectivity and, consequently, behavior. As a result, the SCG can naturally capture widely observed step-like transitions in learning,^96–98^ as well as all-or-none learning effects often observed in conditioning paradigms, such as blocking.^99,100^ Because transition representations can accumulate gradually before edges cross threshold, neural signatures of learning may precede overt behavioral change, consistent with empirical observations.^48^ The same sparsification mechanism explains multimodal individual differences in learned behavior^89,101^ as arising from distinct graph learning trajectories, even when underlying parameters are unimodally distributed.

This sparsification should be understood as an algorithmic abstraction of nonlinear selection rather than as a literal hard cutoff or a specific plasticity mechanism. Any process that selectively retains sufficiently predictive transitions, whether arising from working-memory or attentional gating,^83,102^ will generically induce abrupt changes in effective graph topology. The key distinction is therefore not the threshold itself, but the separation between gradual predictive learning and nonlinear selection of the structure expressed during decision making.

A key computational advantage of the SCG arises from separating predictive learning from online planning. Predictive structure is learned and maintained in the dense transition representation W, whereas decision making operates on the sparse cognitive graph G (see Methods: Computational efficiency). Dense predictive representations such as the SR incur substantial value evaluation costs as the state space grows larger.^3,82,103^ In contrast, the SCG restricts online planning to a sparse directed graph that retains only reliable, task-relevant transitions. Long-horizon consequences can therefore be evaluated by traversing this compact structure rather than by computing dense matrix–vector operations.^104,105^ In many goal-directed tasks, effective planning may preferentially rely on identifying entry and terminal states, without explicit evaluation of all inter-state relationships. This compression supports scalable computation that balances representational economy with flexible behavior^34,35,106^ and is compatible with known limits on working memory capacity^83^ as well as with slot-like accounts of working-memory representations.^16^ These align with evidence that humans favor resource-efficient representations^34–36,107^ and actively prune model branches for planning,^37,38^ and often rely on simplified or discretized approximations of probabilistic structure rather than fully representing graded uncertainty.^108–110^

### Reward, dopamine, and graph formation.

Our analyses suggest that reward facilitates goal-directed graph formation by selectively increasing learning rates for recent reward-predictive transitions in W, thereby increasing the likelihood of threshold crossing for reward-aligned edges in G. We also find evidence that direct dopamine stimulation is consistent with facilitating this process. This interpretation broadly accords with extensive evidence that reward and dopamine enhance learning^68,69,84,85,111,112^ and influence hippocampal and cortical representations during learning.^67,113–116^

The SCG does not posit a single exclusive computational role for dopamine. Instead, it highlights a possible way in which dopaminergic signals can influence which transitions tend to be expressed in internal structure by modulating learning from recent experience. This perspective complements, rather than replaces, established value-based interpretations of dopamine function. A growing body of work suggests that dopamine responses reflect not only reward prediction errors,^49,117^ but also can reflect inferred world structure and may contribute to learning state transitions such as in the SR.^43,52–58,60–66^ In SCG terms, signals like state prediction errors^57,118^ naturally update the transition representation W, thereby potentially influencing downstream graph construction.

Although our analyses focused on reward-based learning, the same principle naturally extends to aversive learning, where dopamine also plays a critical role.^119^ Because avoiding harm can be more behaviorally urgent than obtaining reward, threat or punishment signals may similarly accelerate graph formation, potentially accounting for rapid, one- or few-shot learning observed in aversive contexts.^120^

### Relation to existing cognitive map and retrospective causal inference models.

Recent work on dopamine and distributional reinforcement learning has highlighted asymmetric learning rates for positive and negative prediction errors^121^ and the presence of multiple discounting timescales.^122,123^ Extending the SCG to incorporate multiple discounting rates would yield hierarchical cognitive graphs that express relational structure across multiple temporal scales and levels of abstraction.^124–126^ Such multi-scale representations are common in biological learning systems and confer computational advantages.^125,127–130^

This multi-scale hierarchical formulation connects the SCG to other hippocampal-based models of relational structure, including the Tolman–Eichenbaum Machine, which explicitly supports relational learning across multiple spatial and temporal scales,^4^ and clone-structured causal graphs,^5,131^ particularly if our model is combined with context-dependent graph construction.^132^ Whereas these frameworks emphasize latent structure learning largely independent of reward, the SCG highlights reward-guided compression, offering a complementary account of how hippocampal–prefrontal circuits may balance generalization with goal-directed efficiency.^15,16^

The SCG also aligns with retrospective causal inference models, such as the Adjusted Net Contingency for Causal Relations (ANCCR) model, in which reward receipt appears to trigger retrospective inference.^48^ Within the SCG, accelerated edge learning following reward can give rise to this retrospective appearance even though learning itself remains prospective. Once formed, SCGs are agnostic to prospective or retrospective interpretation, as edges no longer carry graded probabilistic weight. More broadly, by selectively retaining reliable transitions, the SCG encodes directed relational structure that supports counterfactual evaluation and simplifies credit assignment, paralleling core properties of causal graphical models.^133–138^

### Potential neural correlates and predictions.

The SCG is formulated at an algorithmic level, and its neural implementation remains to be established. Nonetheless, the framework suggests potential circuit-level hypotheses and neural predictions. Recent work indicates that the prefrontal cortex supports planning through structured population dynamics, including graph-like representations of task structure with stable start- and goal-related activity,^15,17^ as well as mechanistic recurrent network models in which future trajectories are represented implicitly through neural dynamics.^139^ One possibility consistent with our framework is that hippocampal circuits maintain denser predictive representations resembling successor maps^3^ (corresponding to W), while cortical circuits express a more compact, sparsified graph representation (corresponding to G) that constrains downstream prediction and action.

Because SCGs are directed and often weakly cyclic or acyclic, their spectral structure predicts complementary start- and goal-related population-level signatures reflecting distinct computational roles within the graph. In directed acyclic graphs of the type studied here, right eigenstructure emphasizes entry-point (source) states, whereas left eigenstructure emphasizes terminal (goal) states. Importantly, these signatures are not assumed to correspond to distinct neuron types or stable tuning at the single-neuron level. Instead, they are expected to emerge at the level of neural populations as structured patterns of activity that differentiate states according to their roles in planning and action selection. In this framework, start-related population signatures may contribute to contextual gating and policy selection, whereas goal-related signatures may interface more directly with valuation and outcome-related signals.

Boundary- and context-related neural signals have been widely reported in hippocampal–subicular circuits,^94,140,141^ and suppressing hippocampal–subicular output to prefrontal cortex has been shown to accelerate the emergence of generalized schema representations.^25^ Within the SCG framework, reducing strong context anchoring naturally facilitates the formation of more abstract, transferable cognitive graph structures across tasks. Conversely, the goal-related population signatures predicted by the SCG overlap conceptually with outcome- and goal-related signals reported in prefrontal and frontostriatal systems,^142^ suggesting a potential relationship between graph-defined terminal states and neural signals involved in goal evaluation.

As a speculative extension, if task structure induces sufficient translational invariance across states—for example, through extensive overtraining in highly repetitive trial sequences—grid-like spectral structure may, in principle, also emerge in non-spatial tasks, including the two-step task, for the same mathematical reasons previously described for dense predictive representations in spatial navigation.^3,93^ Although this possibility has not been tested directly in such tasks, low-dimensional grid-like coding has been observed in prefrontal regions across diverse cognitive domains.^26–31^ Within the SCG framework, such patterns are predicted to arise only when graph sparsification preserves sufficient symmetry, highlighting a principled distinction between regimes supporting boundary-localized versus periodic population structure.

### Limitations and future directions.

The inferred graph configurations in the SCG are not intended to map directly onto explicit subjective strategies, nor do we claim that they uniquely determine behavior. Rather, they provide a compact computational description of how learned transition structure can be expressed during decision making under simplifying assumptions. In this work, we adopted a simple Heaviside threshold to illustrate the consequences of sparsification. Alternative nonlinearities, including graded or adaptive thresholds, would likely preserve the core phenomena described here. We assumed that decision making relies primarily on the sparse graph rather than the dense transition representation, allowing us to isolate the impact of the graph structure. More general formulations could allow dense and sparse representations (both W and G) to jointly influence choice, as in mixture or arbitration frameworks.^42,83,143^

Like most reinforcement learning models, we assumed a discrete and stable state space, whereas biological agents must infer latent states, abstractions, and schemas.^144,145^ We did not model replay, which is likely important for offline refinement and reorganization of graph structure.^32,72^ The SCG is specified at an algorithmic level, and its implementation-level model remains to be established. Candidate mechanisms compatible with the framework include STDP-like synaptic plasticity for predictive learning,^146–148^ nonlinear thresholding via dendritic or inhibitory dynamics,^149^ working memory and attentional gating,^83,102^ and synaptic pruning as an offline sparsification process.^150^ Recent energy-based models further demonstrate how local Hebbian plasticity can support learning without backpropagation,^151^ suggesting a possible route for developing neural implementations of SCG-like computations.

Together, these findings suggest that learning compact, reward-aligned cognitive graphs provides a flexible substrate for goal-directed behavior. By integrating reward-modulated predictive learning with sparsification, the SCG provides a mechanistic and interpretable account of how internal goal-directed models develop and guide behavior.

## Methods

### M.1 Computational Model of Sparse Cognitive Graph (SCG)

#### Model overview.

Our framework formalizes how an agent constructs a Sparse Cognitive Graph (SCG) from experience using a reinforcement-learning algorithm. The model maintains two complementary internal representations throughout learning: (1) a dense predictive transition matrix W, which summarizes how often each state predicts its successors under a temporal discount factor, and (2) a sparse directed adjacency matrix G, obtained by thresholding W after each update. Whereas W provides a graded record of experienced transition statistics, G defines the cognitive graph that supports planning, valuation, and action selection. Thresholding yields a compact directed structure that prioritizes reliably experienced transitions and evolves online as experience is acquired. In the formulation used here, decisions are made based on G, rather than directly on W, reflecting the idea that biological agents may rely on simplified internal structure rather than dense predictive matrices.

The Methods are organized to first describe the SCG learning algorithm independently of tasks, followed by task-specific implementations and statistical analyses.

#### Transition representation matrix W.

At each experienced transition $s_{t}\to s_{t+1}$, the model updates a discounted prediction of future successors using a temporal-difference rule anchored on the *observed* next state:

(1) $$\delta_{t}^{TR}\left(s^{\prime }\right)=1\left(s_{t+1}=s^{\prime }\right)+\gamma W\left(s_{t+1},s^{\prime }\right)-W\left(s_{t},s^{\prime }\right),$$

(2) $$W\left(s_{t},s^{\prime }\right)\leftarrow W\left(s_{t},s^{\prime }\right)+\alpha_{t}\delta_{t}^{TR}\left(s^{\prime }\right).$$

This transition representation tracks experienced transitions while propagating predictive strength forward in time via the discount factor γ. In contrast to the classical successor representation (SR), which tracks discounted state occupancy, W remains more localized around experienced one-step transitions and is therefore more amenable to thresholding into a sparse graph. In the limit γ=0, this W update reduces to the FORWARD algorithm.^152^

#### Reward-modulated learning rate.

To allow reward to shape which transitions become embedded in the graph, we permitted learning rates to differ depending on whether a transition was followed by reward:

(3) $$\alpha_{t}=\left\{\begin{array}{ll} \alpha_{\to R}, & \text{if}\;\text{a}\;\text{reward}\;\text{is}\;\text{obtained}\;\text{at}\;\text{time}\;t,\\ \alpha_{\to NoR}, & \text{otherwise} \end{array}\right.$$

We did not impose any ordering on $\alpha_{\to R}$ and $\alpha_{\to NoR}$. If the data support $\alpha_{\to R}>\alpha_{\to NoR}$, then transitions preceding reward naturally accumulate stronger predictive weight in W, biasing the SCG toward reward-aligned structure. This bias is therefore an empirical outcome rather than an a priori assumption.

#### Constructing the sparse cognitive graph.

After each update of W, the agent constructs a sparse directed graph by applying a fixed threshold ζ:

(4) $$G_{ij}=\left\{\begin{array}{ll} 1, & W_{ij}\geq \zeta ,\\ 0, & \text{otherwise.} \end{array}\right.$$

This nonlinear step retains transitions that have accumulated sufficient predictive strength in W. Because thresholding is applied online after each transition, the SCG evolves over time as the agent acquires experience. The adjacency matrix G is then treated as the internal cognitive graph used for valuation and action selection.

In contrast, making decisions directly from W yields an SR–like value readout from a dense predictive representation. Thresholding therefore plays a critical role in testing the hypothesis that behavior relies on sparse cognitive graphs rather than dense predictive maps. Accordingly, we explicitly contrast SCG agents with standard SR agents to assess the necessity of this sparsification step across the tasks analyzed here.

#### Reward estimation and value computation.

Expected reward for each state is learned via a standard delta rule:

(5) $$r\left(s_{t}\right)\leftarrow r\left(s_{t}\right)+\alpha_{r}\left(R_{t}-r\left(s_{t}\right)\right),$$

where $R_{t}$ is the reward observed at time t and $\alpha_{r}$ is the reward-learning rate.

To determine which reward states can be reached from a given state under the current graph, we compute a finite-horizon reachability matrix:^104,105^

(6) $$F=G+G^{2}+\cdots +G^{N},$$

where matrix multiplication and addition are defined over the Boolean semiring, and N is chosen to bound or exceed the graph diameter in the task of interest. In our simulations, where the number of states was small, we computed this quantity explicitly for convenience and binarized the result so that all nonzero entries were set to 1. This gives us the reachability matrix indicating which states can be reached from each other within N steps along the directed graph. After binarization, $F_{ij}=1$ indicates that state i can reach state j along at least one directed path of length at most N.

However, importantly, explicit computation of Eq. (6) via matrix power is not generally required. The same reachability relation can be obtained more efficiently using standard graph traversal algorithms.^105^ Specifically, depth-first search on the directed graph identifies all states that are reachable from a given starting state, implicitly aggregating paths of arbitrary length without explicit matrix operations. This computation scales linearly with the number of graph edges, rather than quadratically with the number of states.

State values can then be computed as

(7) V=Fr,

so that V(s) reflects the aggregate reward that is reachable from s through the learned graph.

This value readout is a deliberately minimal instantiation used to demonstrate how the learned graph can support goal-directed evaluation. When multiple terminal or rewarding states are reachable from a given node, this formulation implicitly aggregates reward across reachable outcomes. Alternative value computations are possible, particularly in graphs with branching structure or differential control over paths, including max-based, policy-weighted, or control-dependent readouts. However, importantly, the central claims of this work do not depend on the specific form of value computation or choice policy. Our contribution of this paper concerns how predictive transition structure is learned and sparsified into a compact directed graph, and how reward and dopamine could bias the formation of that graph. How exactly such learned graphs are subsequently used for valuation and control is an empirical question that will require further targeted experiments to resolve.

#### Relation to the classical successor representation

The classical successor representation (SR) M estimates discounted future state occupancy under the current policy,^82^

(8) $$M\left(s,s^{\prime }\right)=E\left[\sum_{k=0}^{\infty }\;\gamma^{k}1\left(s_{t+k}=s^{\prime }\right)|s_{t}=s\right],$$

and can be learned incrementally via temporal-difference updates. This makes SR a powerful and flexible tool for value computation and has motivated extensive work linking SR-like representations to hippocampal–entorhinal coding.^3,32^

In this work, however, our focus is on constructing a *sparse* directed graph whose edges correspond as directly as possible to learned transitions between states. For this purpose we use the transition–prediction matrix W, which accumulates evidence primarily for observed one-step transitions while still propagating predictive strength via discounting. Entries $W(s,s^{\prime })$ therefore admit a natural interpretation as directed edges in a graph.

This distinction is clearest when γ=0. At this limit, W reduces to the one-step transition matrix, directly reflecting experienced transitions between states. In contrast, the SR collapses to the identity matrix, encoding only trivial self-occupancy and no predictive structure. Thus, W naturally supports graph construction even in regimes where the SR does not provide a meaningful relational representation.

Mathematically, learning W and learning the SR M are closely related. Asymptotically, under this update rule, M≈I+γW, where I is the identity matrix.

We emphasize that this is a modeling choice rather than a limitation of the SR framework: more sophisticated graph-extraction procedures could be applied to SR (for example, based on multi-scale or structured thresholds). The central hypothesis of the SCG is not tied to the specific predictive operator used, but to the nonlinear selection and behavioral expression of predictive transitions in a sparse directed graph. We adopt W in this paper because, for the SCG mechanism studied here, it provides a simple and transparent route from experience to a sparse, directed cognitive graph.

### Computational efficiency.

In SCG, downstream inference and value computation operate on a sparse cognitive graph rather than on dense predictive matrices. Let $N_{\text{SCG}}$ denote the number of nodes in the learned sparse cognitive graph and |E| the number of directed edges. Storage of the graph scales as O(|E|), with $|E|\ll N_{\text{SCG}}^{2}$ in structured environments. Reachability-based inference can be implemented using standard graph traversal algorithms, yielding computational cost $O\left(|E|+N_{\text{SCG}}\right)$.^104,105^

By contrast, dense predictive representations such as the transition representation W or the SR operate over the full state space. Let $N_{SR}$ denote the number of states represented in these dense models. Such representations require $O\left(N_{SR}^{2}\right)$ storage and compute values via dense linear operations on the reward vector, incurring $O\left(N_{SR}^{2}\right)$ computational cost per evaluation.^3,82^

Thus, SCG yields a reduction in computational cost through two distinct mechanisms: (i) sparsification of relational structure ($|E|\ll N_{\text{SCG}}^{2}$), and (ii) compression of the state representation ($N_{SCG}\ll N_{SR}$).

### M.2 Alternative models.

To situate SCG within the broader reinforcement-learning literature, we implemented four standard alternatives: (1) the classical successor representation (SR), (2) a purely model-free temporal-difference (TD) learner, (3) a fully model-based (MB) planner, and (4) a hybrid MB/MF mixture. All alternative models were evaluated on the same revaluation and two-step tasks and analyzed using the same revaluation-score and stay-probability measures.

### Classical successor representation (SR).

The successor representation (SR) matrix M encodes discounted future state occupancy,

(9) $$M\left(s,s^{\prime }\right)=E\left[\sum_{k=0}^{\infty }\;\gamma^{k}1\left(s_{t+k}=s^{\prime }\right)|s_{t}=s\right].$$

We used a standard one-step temporal-difference update that enforces the SR Bellman equation,

(10) $$M\left(s_{t},s^{\prime }\right)\leftarrow M\left(s_{t},s^{\prime }\right)+\alpha_{M}\left(1\left(s_{t}=s^{\prime }\right)+\gamma M\left(s_{t+1},s^{\prime }\right)-M\left(s_{t},s^{\prime }\right)\right),$$

which includes an explicit identity term reflecting current-state occupancy. In parallel, expected rewards were learned via a delta rule,

(11) $$r\left(s_{t+1}\right)\leftarrow r\left(s_{t+1}\right)+\alpha_{r}\left(R_{t}-r\left(s_{t+1}\right)\right).$$

State values were then computed as

(12) $$V(s)=\sum_{s^{\prime }}M\left(s,s^{\prime }\right)r\left(s^{\prime }\right).$$

Compared to SCG, the SR encodes a dense predictive map of discounted state occupancy, rather than a sparse directed graph of transitions.

### Model-free TD learner.

The model-free learner updates scalar state values via:

(13) $$V\left(s_{t}\right)\leftarrow V\left(s_{t}\right)+\alpha \left(R_{t}+\gamma V\left(s_{t+1}\right)-V\left(s_{t}\right)\right),$$

with no explicit transition model. This makes it unable to propagate revaluation through unexperienced starting states.

### Model-based reinforcement learning.

We applied a standard form of MB agent to the two-step task, where asymptotic knowledge of the transition model is given to the agent.^42,66^ To understand the variability of learning, we additionally applied a version of the MB agent that learns the transition model T^152^.^89^ After each observed transition $s_{t}\to s_{t+1}$, the corresponding entry in T is incremented and the row is renormalized to form empirical conditional probabilities.^152^ Given T and learned reward estimates, values are obtained by tabular policy evaluation under the learned dynamics.

### Hybrid MB/MF mixture.

In the revaluation experiment, the hybrid agent estimates model-based and model-free values, to estimate the respective ratings (see below), which are combined following the expression :

(14) $$V_{Hybrid}=wR_{MB}+(1-w)R_{MF},$$

with weighting parameter w.

### M.3 Task descriptions.

We evaluated the model on previously published behavioral tasks and datasets.

### Human reward and transition revaluation tasks^89^

We modeled the reward and transition revaluation tasks of Momennejad et al.^89^ using a six-state environment {A,B,C,D,E,F}. States C and F served as terminal states associated with fixed monetary outcomes of 10 (bonus reward) and 1 (baseline outcome), respectively. During initial learning, agents experienced the deterministic structure

A→B→C→Reward,D→E→F→Baselineoutcome.

Each sequence was presented 20 times, matching the original experiment. In the experiments, participants reported their relative preference between the two starting states A and D using a continuous rating scale. These ratings were stored as values that lie between 0 and 1.

In the **reward revaluation** condition, terminal outcomes were swapped while the transition structure was preserved,

B→C→Baselineoutcome,E→F→Reward,

again for 20 presentations; the initial states A and D were omitted during revaluation.

In the **transition revaluation** condition, the second-step transitions were swapped while terminal outcomes remained unchanged,

B→F→Baselineoutcome,E→C→Reward.

After the revaluation stage, participants were asked to report their relative preference between the two starting states A and D. The experimental revaluation score was defined as the difference between the post- and pre-relearning preference ratings.^89^

### Human two-step task.^42^

The standard human two-step task^42^ comprised a first-stage choice between two options, followed by probabilistic transitions to one of two second-stage states. First-stage choices (A or B) led to second-stage states (C or D) with common and rare transitions: P(C|A)=0.7,P(D|A)=0.3, P(C|B)=0.3,P(D|B)=0.7. At the second stage, participants chose between two cues. Reward probabilities for each cue drifted independently over time according to bounded random walks with Gaussian increments (step size σ=0.025) and reflecting boundaries [0.25, 0.75], updated every trial.

### Mouse two-step task and optogenetic stimulation.^66^

We reanalyzed the publicly available mouse two-step task dataset published in.^66^ Here we summarize the task structure and stimulation protocols relevant for the present analyses. Mice initiated each trial by poking a lit center port and then made a first-stage choice between left and right ports. On 75% of trials both options were available (free-choice), whereas on 25% only a single option was available (forced-choice). Each choice led with 80% (common) vs. 20% (rare) probability to one of two second-step ports (up/down), signaled by distinct auditory and visual cues; the mapping from first- to second-step states was fixed within animals and counterbalanced across animals. Entering the active second-step port triggered a second-step-specific outcome cue indicating reward (either up- or down-reward cue) or no reward (a shared no-reward cue). Reward probabilities at second-step ports varied by block. Biased blocks (80%/20%) ended when an exponentially weighted moving average of correct choices (time constant of 8 free-choice trials) exceeded 75%; balanced blocks (50%/50%) lasted 20–30 trials before pseudo-random switching. Trials ended with a 2–4 s intertrial interval.

Optogenetic stimulation was delivered through implanted optical fibers targeting dopaminergic neurons. In separate experimental sessions, stimulation was applied either at the second-step onset or at the outcome onset. In each session, stimulation occurred on 25% of trials with two constraints: (i) the following trial was always free-choice, and (ii) at least two non-stimulated trials followed each stimulation event. Twelve DAT-Cre mice were tested (5 YFP controls and 7 ChR2 experimental animals). Further procedural details are provided in.^66^

### M.4 Model applications to tasks

#### Human reward and transition revaluation tasks.

We applied our model to,^89^ where transition learning rates differed between trials yielding the bonus ($10) and baseline outcomes ($1), with the baseline outcome treated as a reference level such that learning-rate modulation reflected the presence ($\alpha_{\to R}$) versus absence ($\alpha_{\to NoR}$) of additional reinforcement.

To characterize qualitative regimes of SCG behavior, we computed phase diagrams over model parameters. To generate Figure 2IJ, we varied the baseline transition learning rate $\alpha_{\to NoR}$ and the discount factor γ sampled logarithmically from 10^−2^ to 1, with other parameters fixed at $\alpha_{\to R}=0.4,\;\alpha_{r}=0.9$, and ζ=0.7. For each parameter set, we simulated the full reward or transition revaluation protocol and computed values for the two starting states before revaluation (*pre*) and after revaluation (*post*). Following,^89^ the model computes a latent preference signal for D over A at time τ∈{pre,post} as the value difference $\Delta V_{\tau }=V_{\tau }(D)-V_{\tau }(A)$. To mirror the bounded behavioral rating, the reported preference was modeled by mapping this latent signal through a Gaussian cumulative distribution function (probit link), yielding a continuous rating between 0 and 1, following.^89^ The generated ratings, $R_{\tau }$ were used to define the revaluation score within the model as the change in preference,

(15) $$RS=R_{post}-R_{pre}.$$

Thus, RS=0 indicates no change in preference, RS>0 indicates a shift toward D (reversal relative to an initial preference).

For the parameter ranges considered here, the model satisfied $V_{\text{pre}}(A)>V_{\text{pre}}(D)$ at the end of initial learning, consistent with the data in.^89^ We therefore characterized qualitatively distinct revaluation behavioral regimes based on the relative ordering of starting-state values after relearning: (i) $V_{\text{post}}(A)>V_{\text{post}}(D)$, indicating that the initial preference was preserved; (ii) $V_{\text{post}}(A)<V_{\text{post}}(D)$, indicating a reversal of preference; and (iii) $V_{\text{post}}(A)\approx V_{\text{post}}(D)$, indicating indifference between the two starting states.

To generate multimodal preference distributions (Figure 2EFGH), we drew parameters from unimodal Gaussian distributions centered near phase boundaries. For reward revaluation, we set $\mu_{\alpha }=0.055$ and $\mu_{\gamma }=0.68$, and for transition revaluation, $\mu_{\alpha }=0.042$ and $\mu_{\gamma }=0.68$. Variances were set to 0.001 for both parameters, with zero off-diagonal covariance. To mirror the behavioral measure (a noisy preference rating bounded between 0 and 1), following,^89^ we assumed that the revaluation score on each trial was sampled from a Gaussian distribution with mean equal to the model-predicted revaluation score (RS), with noise σ=0.15 for reward revaluation and σ=0.1 for transition revaluation.

We also simulated alternative models to generate the phase diagrams shown in Fig. S2. For the mixture of model-free and model-based reinforcement learning, the weighting parameter w∈[0,1] was drawn from a Gaussian distribution 𝒩(0.5,0.18) and clipped to the unit interval.

#### Human two-step task.

To test whether the SCG reproduces canonical stay-probability signatures in the human two-step task, we simulated agents interacting with the task structure described above^42^ and analyzed behavior using conditional stay probabilities as a function of previous trial outcome and transition type.

We modeled a first-stage action a as deterministically selecting a distinct first-stage decision state $s_{a}$. State values were computed from the current sparse cognitive graph, and first-stage action values were defined as

(16) $$Q(a)=V\left(s_{a}\right)+\beta_{\text{stay}}B(a),\;a\in \{A,B\},$$

where $s_{a}$ denotes the state associated with action $a,\;V\left(s_{a}\right)$ is the value of that state computed from the SCG, and B(a)=1 if action a was chosen on the previous trial and 0 otherwise, capturing a standard stay bias.^42,91^ Choices followed a softmax policy,

(17) $$P(a)=\frac{exp(Q(a)/T)}{\sum_{b}\;exp(Q(b)/T)},$$

with temperature T controlling choice stochasticity. Second-stage choices were modeled as ideal selections of the option with the higher reward probability, while reward vectors, including of those at second-stage states (C or D), were learned via a standard delta rule. On each trial, the agent chose between A and B using the softmax policy described above and updated W,G, and r according to the SCG learning rules at the experienced transitions.

We conducted a grid search over parameters targeting the conditional stay probabilities (transition × outcome) reported in.^42^ Results presented in Figure 3 were generated from simulations using a representative parameter set that reproduced the canonical stay-probability interaction with $\alpha_{\to R}=0.6$, $\alpha_{\to NoR}=0.3$, $\alpha_{r}=0.9$, γ=0.6, T=0.5, ζ=0.6, and $\beta_{\mathrm{stay}}=0.6$.

#### Mouse two-step task and optogenetic stimulation.

We applied the SCG model to the mouse two-step task dataset reported in,^66^ using a hierarchical Bayesian modeling framework. The model state space was defined to reflect the animals’ experienced task structure:

𝒮={leftchoice,rightchoice,upcue,downcue,up−rewardcue,down−rewardcue,no−rewardcue}.

Choices (left vs. right) were modeled using the same action–state mapping (Eq. (16)) and choice policy (Eq. (17)) as in the human two-step task. Following prior analyses of this dataset^66^ and related modeling work,^83,91^ we assumed a *forgetful* reward representation, in which all elements of the reward vector were updated at a common rate, regardless of whether the corresponding state was visited. A non-forgetting variant of the SCG model was also evaluated but performed worse in terms of model evidence assessed by iBIC.

To estimate conditional stay probabilities (Fig. 4C), we simulated the SCG with fitted parameters under the same experimental conditions as the behavioral data. Transition learning-rate estimates shown in Fig. 4D were taken directly from session-by-session MAP estimates.

We modeled optogenetic stimulation in ChR2 animals at outcome timing as a boost in transition learning rates. We allowed distinct transition learning rates on stimulated trials, $\alpha_{s,\to R}$ and $\alpha_{s,\to NoR}$, in addition to the baseline learning rates $\alpha_{\to R}$ and $\alpha_{\to NoR}$ governing non-stimulated trials. Model predictions shown in Fig. 4G were generated using a representative parameter set ($\alpha_{\to R}=0.2$, $\alpha_{\to NoR}=0.1$, $\alpha_{r}=0.5$, γ=0.4, T=0.3, ζ=0.6, $\beta_{\text{stay}}=0.4$, $\alpha_{s,\to R}=0.7$, $\alpha_{s,\to NoR}=0.3$), chosen to illustrate the qualitative effects predicted by the model.

For comparison, we additionally fit several alternative models to the mouse data, including: (i) an SCG variant with a single transition learning rate ($\alpha_{\to R}=\alpha_{\to NoR}$), (ii) an SCG variant without reward forgetting, (iii) the winning reinforcement-learning model reported in the original study (model-based reinforcement learning with asymmetric reward learning and forgetting),^66^ as well as standard successor representation, model-free, and model-based reinforcement-learning agents. Comparative results are shown in Fig. S4.

#### Hierarchical Bayesian model fitting.

To robustly estimate model parameters h, we performed a hierarchical Bayesian random-effects analysis^37,91^ separately for each animal. In this framework, the (appropriately transformed) parameter vector $h_{i}$ for experimental session i is treated as a random draw from a Gaussian population distribution with mean and covariance $\theta =\left\{\mu_{\theta },\Sigma_{\theta }\right\}$. The subject-level parameter θ is estimated by maximizing the marginal likelihood

(18) $$\begin{array}{l} \theta^{ML}\;\approx \arg\underset{\theta }{max}\;p(D|\theta )\\ \;=arg\;\underset{\theta }{max}\;\left\{\prod_{i=1}^{N}\;\int dh_{i}p\left(D_{i}|h_{i}\right)p\left(h_{i}|\theta \right)\right\}. \end{array}$$

We optimized θ using an approximate Expectation–Maximization (EM) procedure. In the E-step of iteration k, a Laplace approximation yields

(19) $$m_{i}^{k}\approx arg\;\underset{h}{max}\;\left\{p\left(D_{i}|h\right)p\left(h|\theta^{k-1}\right)\right\},$$

(20) $$p\left(h_{i}|D_{i}\right)\approx 𝒩\left(m_{i}^{k},\Sigma_{i}^{k}\right),$$

where $m_{i}^{k}$ is the maximum-a-posteriori (MAP) estimate and $𝒩\left(m_{i}^{k},\Sigma_{i}^{k}\right)$ denotes a multivariate normal distribution with mean $m_{i}^{k}$ and covariance $\Sigma_{i}^{k}$, obtained from the inverse Hessian evaluated at $m_{i}^{k}$.

In the M-step, population parameters are updated as

(21) $$\mu_{\theta }^{k+1}=\frac{1}{N}\sum_{i=1}^{N}\;m_{i}^{k},$$

(22) $$\Sigma_{\theta }^{k+1}=\frac{1}{N}\sum_{i=1}^{N}\;\left(m_{i}^{k}m_{i}^{kT}+\Sigma_{i}^{k}\right)-\mu_{\theta }^{k+1}\mu_{\theta }^{k+1T}.$$

For simplicity, we assumed a diagonal covariance matrix $\Sigma_{\theta }$, corresponding to independent population-level effects.

When fitting the model to mouse data,^66^ model parameters were estimated by maximizing the log-likelihood over all free-choice trials.

#### Model comparison.

Models were compared using the integrated Bayesian Information Criterion (iBIC).^37,91^ The marginal log likelihood of model M is given by

(23) logp(D∣M)=∫dθp(D∣θ)p(θ∣M)

(24) $$\approx \log p\left(D|\theta^{ML}\right)-\frac{1}{2}\left|M\right|\log\left|D\right|=-\frac{1}{2}iBIC,$$

where |M| denotes the number of fitted population-level parameters and |D| is the total number of observed choices across all subjects.

The marginal likelihood term $log\;p\left(D|\theta^{ML}\right)$ was computed by integrating out individual-level parameters:

(25) $$log\;p\left(D|\theta^{ML}\right)\;=\sum_{i}\;log\int \;dh\;p\left(D_{i}|h\right)p\left(h|\theta^{ML}\right)$$

(26) $$\approx \sum_{i}\;\log\left(\frac{1}{K}\sum_{j=1}^{K}\;p\left(D_{i}|h^{j}\right)\right),$$

where the integral was approximated by Monte Carlo averaging over K samples $h^{j}$ drawn from the fitted prior $p\left(h|\theta^{ML}\right)$.

### M.5 Behavioral data analyses of the impact of optogenetic stimulation^66^

We focused on sessions in which optogenetic stimulation was delivered at the time of outcome onset and restricted analyses to balanced reward blocks (50%/50%) to minimize the influence of blockwise reward biases. For each free-choice trial t, we computed the probability of repeating the same first-stage choice on the next free-choice trial $t\;+\;1,\;P(\left.{stay}_{t+1}\right)$, conditioned on second-step transition (common vs. rare), outcome (reward vs. no reward), and stimulation (on vs. off). Because stimulation was experimentally constrained to be followed by at least two non-stimulated trials, these intervening trials were excluded; analyses were restricted to free-choice t and t+1.

For descriptive comparisons, we contrasted $\left.{P(stay}_{t+1}\right)$ following rare–reward trials with vs. without stimulation. Statistical significance of stimulation effects was assessed using permutation tests implemented in Python (SciPy). To quantify the joint influence of group and trial events, we fit a mixed-effects logistic regression,

$${\text{Stay}}_{t+1}∼\text{Group}\;*\;\text{Stim}\;*\;\text{Transition}\;*\;\text{Outcome},$$

where Group (ChR2 vs. YFP), Stim (on vs. off), Transition (common vs. rare), and Outcome (reward vs. no reward) were entered as fixed effects, and mouse identity was modeled as a random effect. Analyses were restricted to free-choice trials, and permutation-based tests were specifically applied to compare stimulated vs. non-stimulated rare–reward trials.

### M.6 Spectral analysis of SCG

To characterize low-dimensional structure in the learned SCG, we performed spectral analyses of the directed adjacency matrix G. Because G is generally non-symmetric, we analyzed both right eigenvectors, defined by Gv=λv, and left eigenvectors, defined by $u^{⊤}G=\lambda u^{⊤}$ (equivalently, eigenvectors of $G^{⊤}$). Right eigenvectors emphasize structure associated with outgoing connectivity, whereas left eigenvectors emphasize structure associated with incoming connectivity. Eigenvectors associated with zero eigenvalues correspond to the null spaces of G and $G^{⊤}$. These null spaces identify directions in state space that are not propagated under repeated application of the adjacency operator. In acyclic or sparsely connected graphs, such modes are typically associated with states lacking outgoing (terminal) or incoming (initial) connectivity, reflecting boundary structure or net-zero flow in the learned graph.

Spectral modes were examined for localization and periodicity across states under different task conditions. In particular, we compared eigenstructure across settings that differed in graph sparsity and cyclicity, including cases in which transitions through an inter-trial interval (ITI) state were weak or absent, and cases in which training induced recurrent transitions linking reward states to subsequent trial onset.

For reference, we also performed the same analyses on the dense predictive representation W. In contrast to the sparsified adjacency matrix G, the matrix W encodes graded long-horizon transition statistics accumulated across multiple paths. As a result, its spectral structure reflects averaged predictive flow rather than discrete reachability boundaries. In directed but non-cyclic task configurations, eigenmodes of W can exhibit partial localization at source or sink states. This arises because, in absorbing or strongly directed systems, the dominant eigenstructure of a predictive operator is shaped by imbalance between incoming and outgoing predictive mass, leading to accumulation near terminal states. However, because W retains contributions from many indirect transitions, these modes are typically smoother and less sharply localized than those obtained from the thresholded graph G.

When task dynamics induce approximate translational invariance through recurrent transitions, the spectrum of W is dominated by low-frequency modes associated with repeated structure, yielding periodic eigenmodes analogous to those previously described for the successor representation in spatial random walk settings.^3^ In this regime, spectral modes primarily reflect global predictive geometry rather than discrete start or terminal roles. By contrast, sparsification into G suppresses weak and indirect transitions, accentuating boundary structure and emphasizing directed reachability relevant for planning and valuation.

All spectral analyses were performed directly on adjacency matrices rather than graph Laplacians,^3^ which are more appropriate for undirected or diffusion-based analyses, as the learned representations were explicitly directed. This ensured that extracted eigenmodes reflected the directed reachability structure underlying computation in the SCG.

### M.7 Simulations of grid- and flag-like representations

To visualize boundary-localized eigenmodes in a cognitive task, we used the learned G from the mouse two-step environment with seven states and threshold ζ=0.6. We computed left and right eigenvectors of G were computed and visualized on a schematic representation of the task, using Gaussian interpolation for display purposes.

To examine conditions under which periodic structure can emerge in a non-spatial task, we extended the state space with an inter-trial interval (ITI) state that returned to left or right with equal probability, P(left|ITI)=P(right|ITI)=0.5, and used lower threshold ζ=0.5. This produced a cyclic graph spanning trial onset, transition, outcome, ITI, and subsequent trial onset. Spectral decomposition of G in this augmented graph yielded periodic eigenmodes along the trial sequence, analogous to grid-like structure in abstract state spaces.

At higher sparsification thresholds, the adjacency matrix G no longer preserved cyclic structure to support periodic eigenmodes. In these cases, however, periodic structure remained evident in spectral decompositions of the dense transition representation W, reflecting its sensitivity to translational symmetry in the underlying transition statistics even when such structure is suppressed by graph sparsification.

For comparison, we simulated a 12 × 12 spatial grid world in which agents moved uniformly in up to four directions with reflective boundaries.

## Supplementary Material

Supplement 1
