# Supplementary material for: Building Goal-Directed Cognitive Graphs
Source: bioRxiv. 2026 Mar 5:2026.02.18.706628. Originally published 2026 Feb 19. Preprint. [Version 2] doi: 10.64898/2026.02.18.706628 (PMC12934822; doi:10.64898/2026.02.18.706628)
Supplement: Supplementary file 1 [file NIHPP2026.02.18.706628v2-supplement-1.pdf]

bioRxiv preprint doi: https://doi.org/10.64898/2026.02.18.706628; this version posted March 5, 2026. The copyright holder for this preprint (which was not certified by peer review) is the author/funder, who has granted bioRxiv a license to display the preprint in perpetuity. It is made available under a CC-BY 4.0 International license.

Supplementary Figures

bioRxiv preprint doi: https://doi.org/10.64898/2026.02.18.706628; this version posted March 5, 2026. The copyright holder for this preprint (which was not certified by peer review) is the author/funder, who has granted bioRxiv a license to display the preprint in perpetuity. It is made available under a CC-BY 4.0 International license.

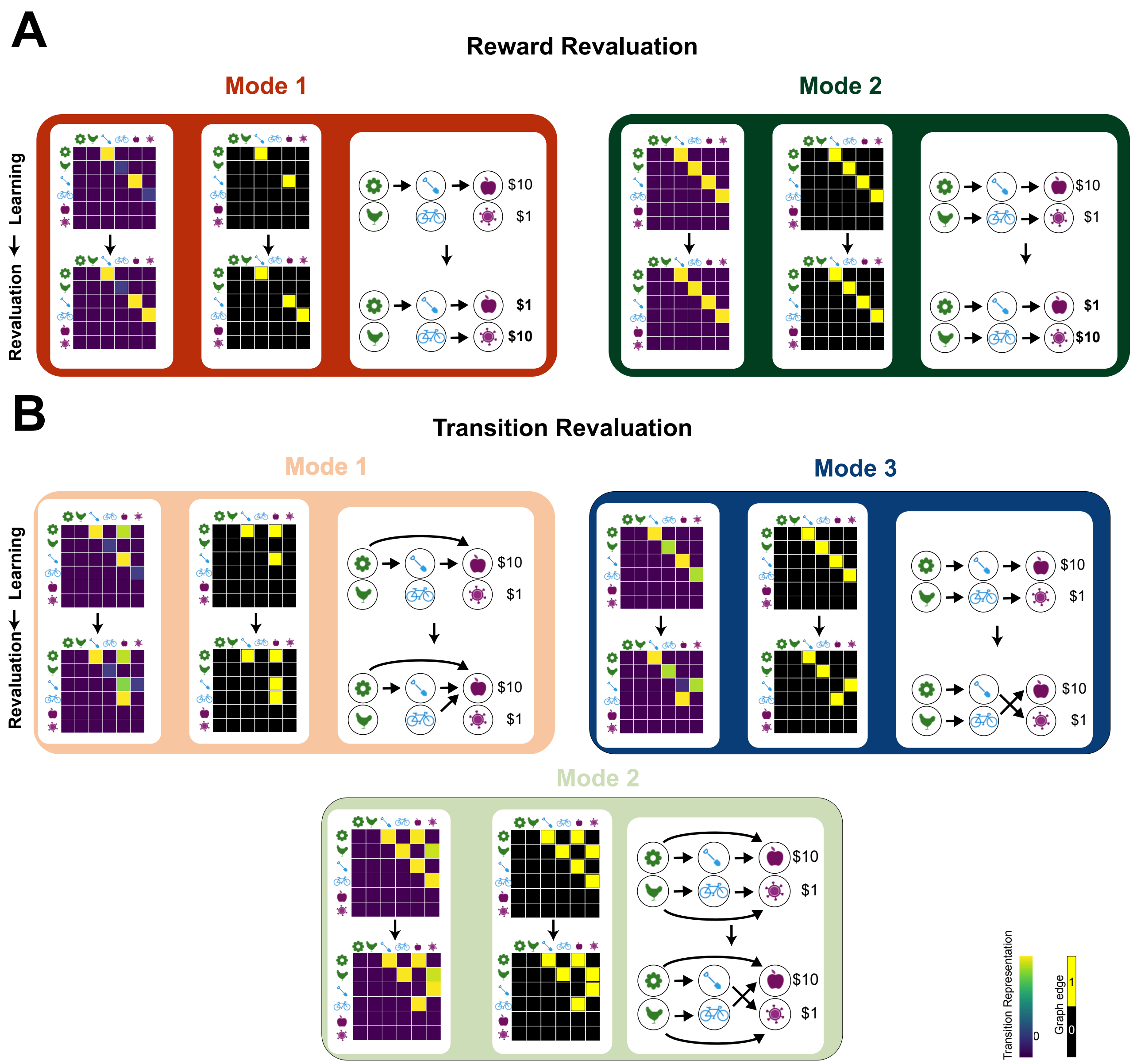

Figure S1: Transition representation W and thresholded adjacency matrix G that underlie multimodal distributions in **(A)** reward revaluation and **(B)** transition revaluation.

bioRxiv preprint doi: https://doi.org/10.64898/2026.02.18.706628; this version posted March 5, 2026. The copyright holder for this preprint (which was not certified by peer review) is the author/funder, who has granted bioRxiv a license to display the preprint in perpetuity. It is made available under a CC-BY 4.0 International license.

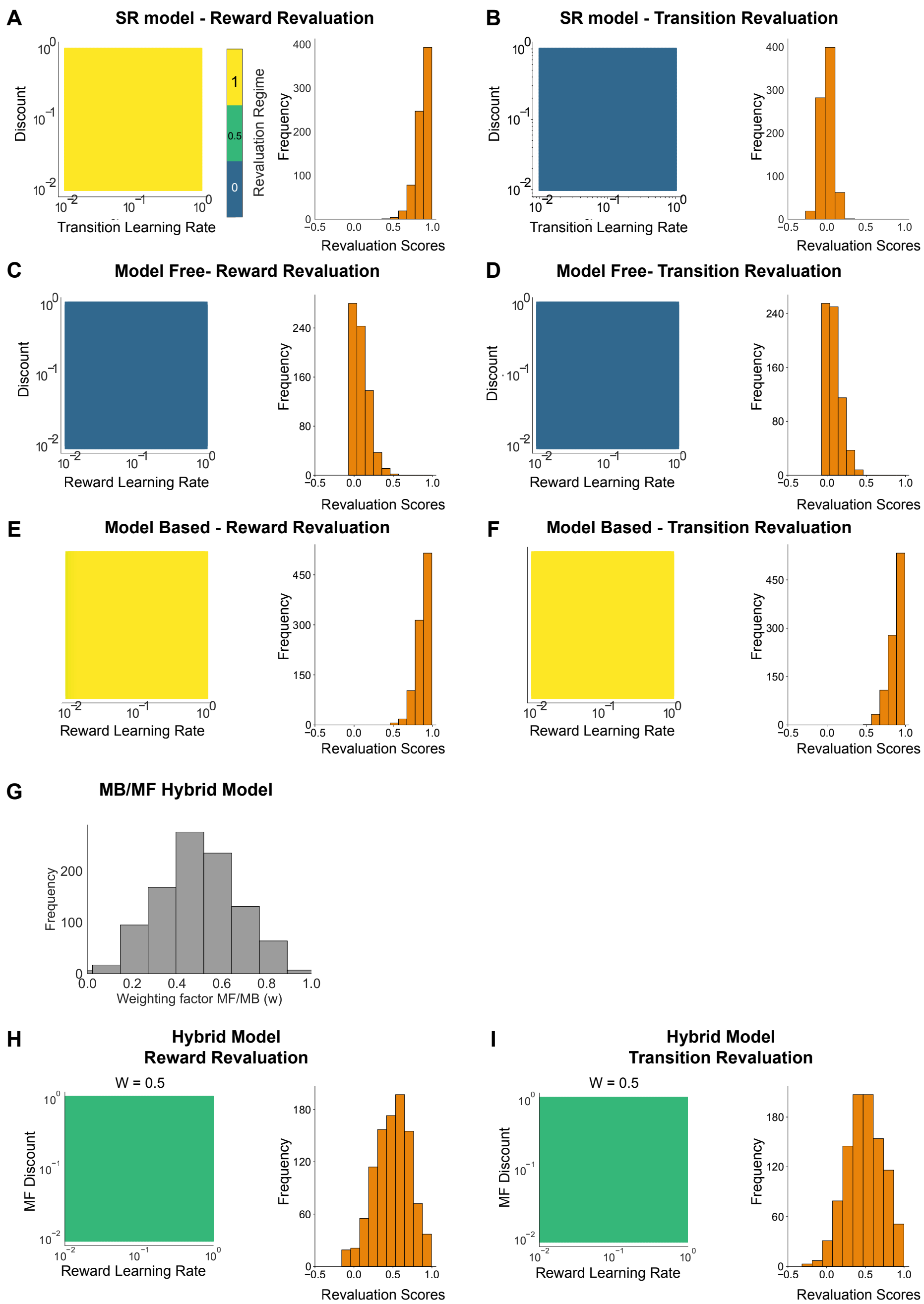

Figure S2: Alternative models fail to capture multimodal behavioral regimes in reward and transition revaluation tasks.[48] (**A**) and (**B**). Successor representation (SR) model. Phase diagrams (left) and predicted revaluation score distributions (right). In the reward revaluation task, the SR model predicts a uniform change in preference for the initial states across parameter regimes (**A**, left), yielding a unimodal distribution of revaluation scores centered at 1 (complete change; **A**, right). In contrast, in the transition revaluation task, the SR model predicts no change in preference for the initial states regardless of parameters (**B**, left), resulting in a unimodal distribution centered at 0 (no change; **B**, right). (**C**) and (**D**). Model-free reinforcement learning (temporal-difference learning). The model predicts no change in preference for both reward and transition revaluation tasks across parameter regimes. (**E**) and (**F**). Model-based reinforcement learning. A transition model combined with forward planning predicts complete reversal of preferences in both tasks. (**G**). Hybrid model-based/model-free (MB/MF) models with a distributed weighting parameter between model-based and model-free controls (left) fail to reproduce the multimodal distributions observed in human behavior for either reward or transition revaluation (right).

bioRxiv preprint doi: https://doi.org/10.64898/2026.02.18.706628; this version posted March 5, 2026. The copyright holder for this preprint (which was not certified by peer review) is the author/funder, who has granted bioRxiv a license to display the preprint in perpetuity. It is made available under a CC-BY 4.0 International license.

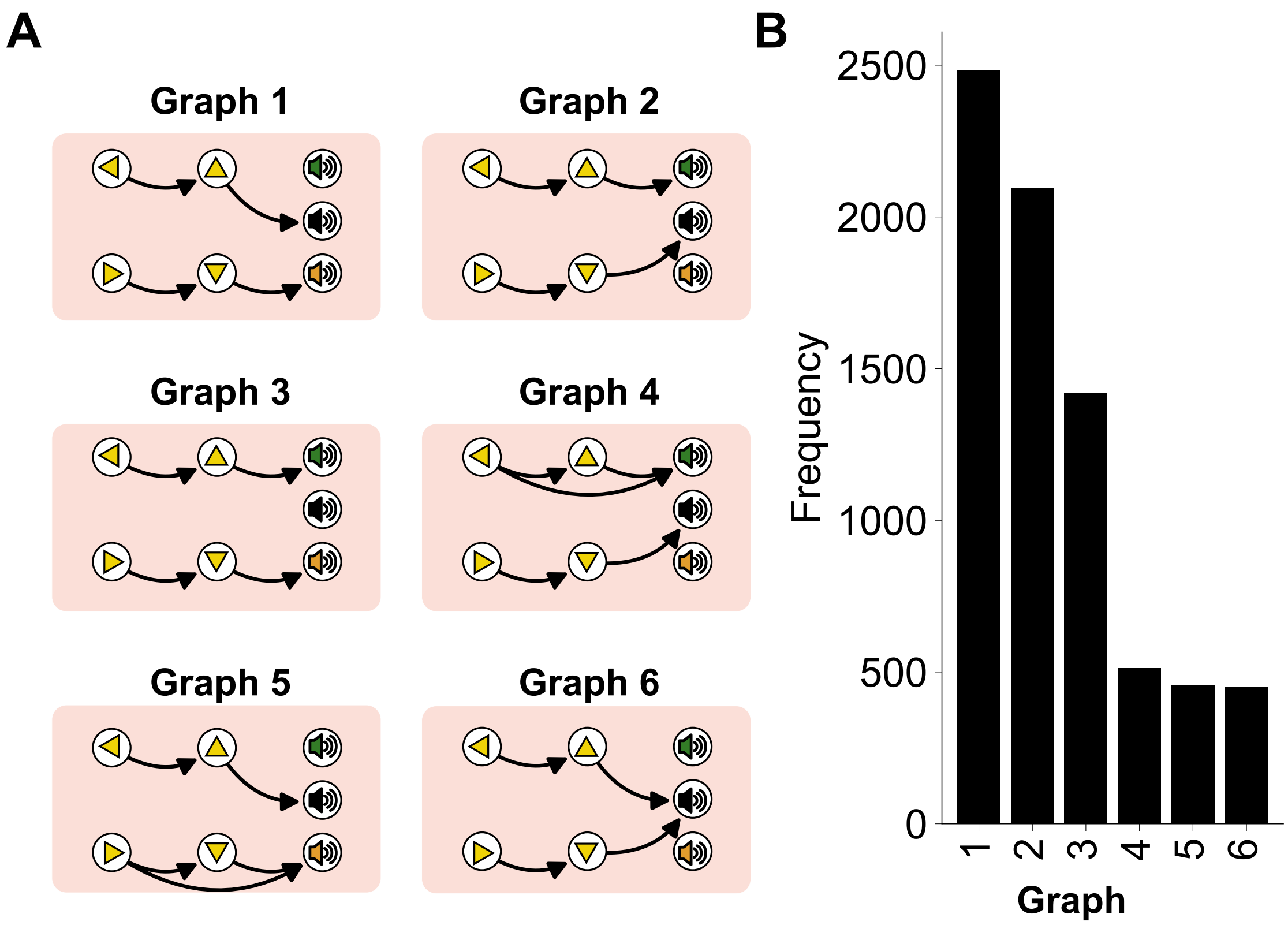

Figure S3: Graph configuration frequencies in the mouse two-step task. (**A**). Most frequently occurring graph configurations. (**B**). Histogram of graph configuration frequencies for the representative animal.

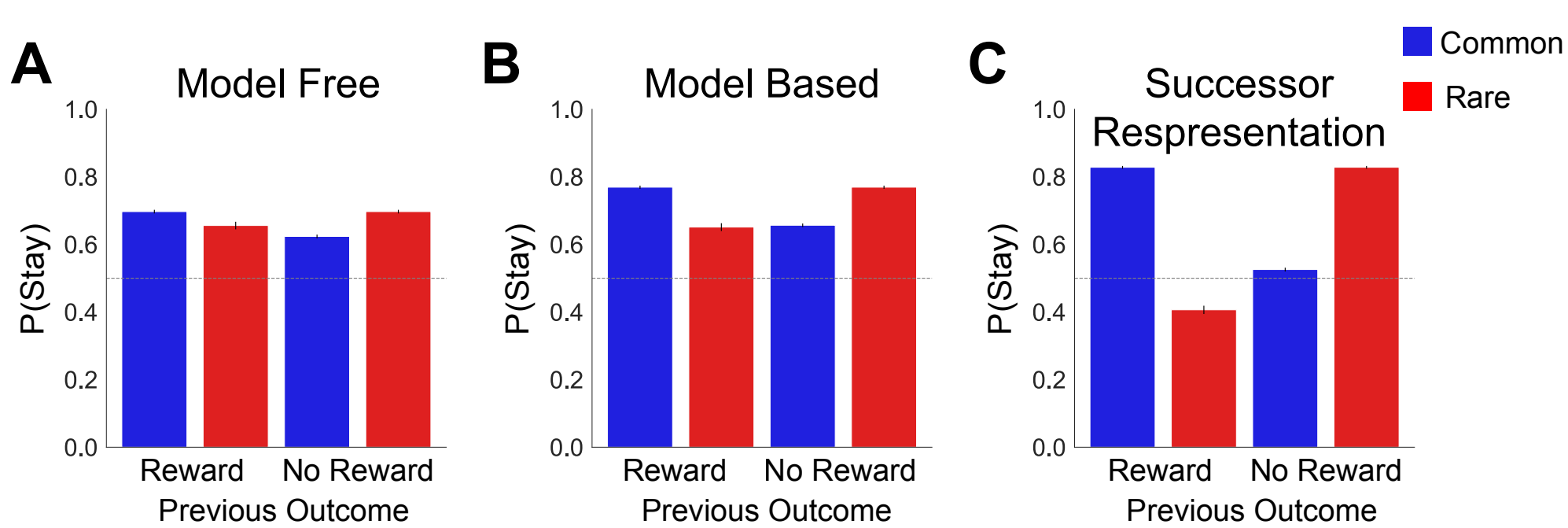

Figure S4: Alternative models fail to reproduce mouse two-step behavior. (**A**) Standard model-free temporal difference learning model. (**B**) Standard model-based reinforcement learning model. (**C**) Standard SR model. Error bars indicate s.e.m. across simulations.

bioRxiv preprint doi: https://doi.org/10.64898/2026.02.18.706628; this version posted March 5, 2026. The copyright holder for this preprint (which was not certified by peer review) is the author/funder, who has granted bioRxiv a license to display the preprint in perpetuity. It is made available under a CC-BY 4.0 International license.

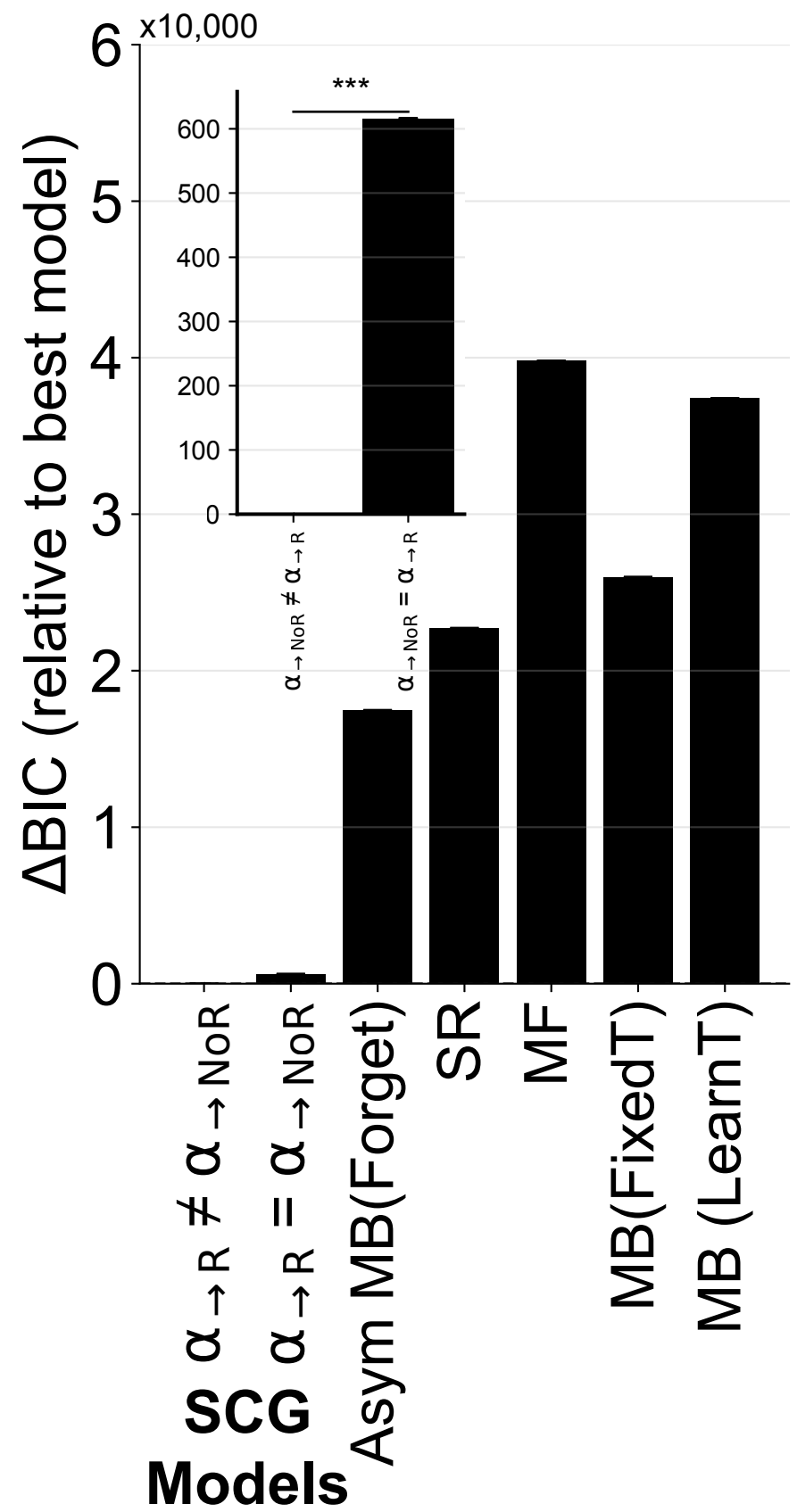

Figure S5: Model comparison for mouse two-step task.[50] Integrated Bayesian Information Criterion (iBIC) scores are shown for models fit using a hierarchical Bayesian approach. The SCG model with separate transition learning rates for rewarded and unrewarded outcomes ($\alpha_{\rightarrow \mathrm{R}}$ and $\alpha_{\rightarrow \mathrm{NoR}}$) outperformed the SCG model with a single transition learning rate ($\alpha_{\rightarrow \mathrm{R}} = \alpha_{\rightarrow \mathrm{NoR}}$), as well as the winning model of previous analysis (model-based RL with asymmetric learning and forgetting[50]) standard SR, standard model-free, and standard model-based agents with fixed transition structure and learned transition structure. The inset shows a zoomed view of the iBIC differences between SCG variants. Lower iBIC values indicate better model fit. Error bars indicate the cross-sample mean ± s.e.m. (4,000 bootstrap samples from the posterior distribution)

bioRxiv preprint doi: https://doi.org/10.64898/2026.02.18.706628; this version posted March 5, 2026. The copyright holder for this preprint (which was not certified by peer review) is the author/funder, who has granted bioRxiv a license to display the preprint in perpetuity. It is made available under a CC-BY 4.0 International license.

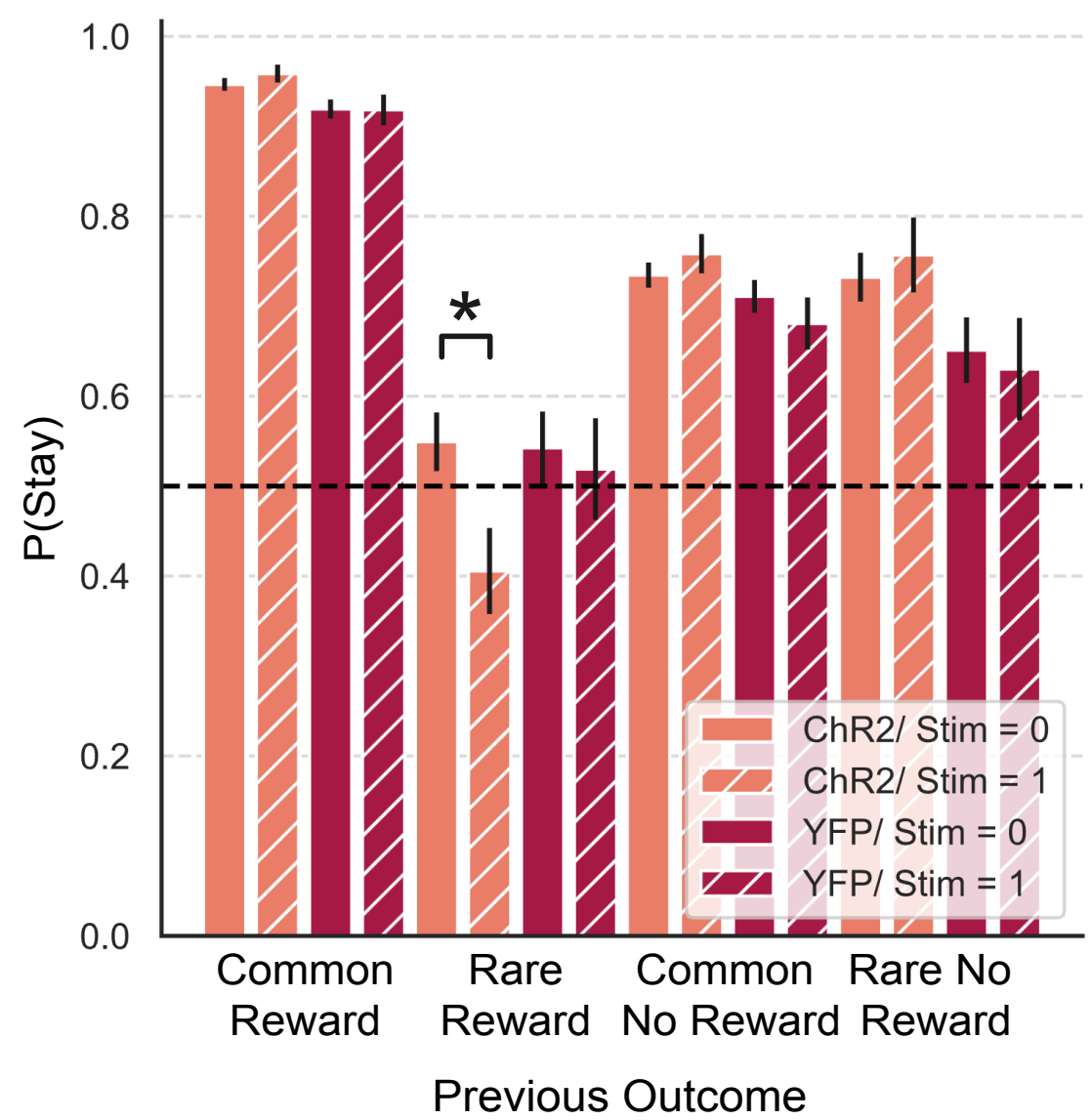

Figure S6: Optogenetic stimulation effects on conditional stay probability.[50] Optogenetic stimulation at outcome timing in the mouse two-step task is associated with differences in subsequent choice behavior following rare transitions. Choice probabilities are shown for ChR2 (orange) and YFP control (red) groups, comparing trials with stimulation (shaded bars) and without stimulation (solid bars) across transition and outcome conditions. Error bars indicate s.e.m..

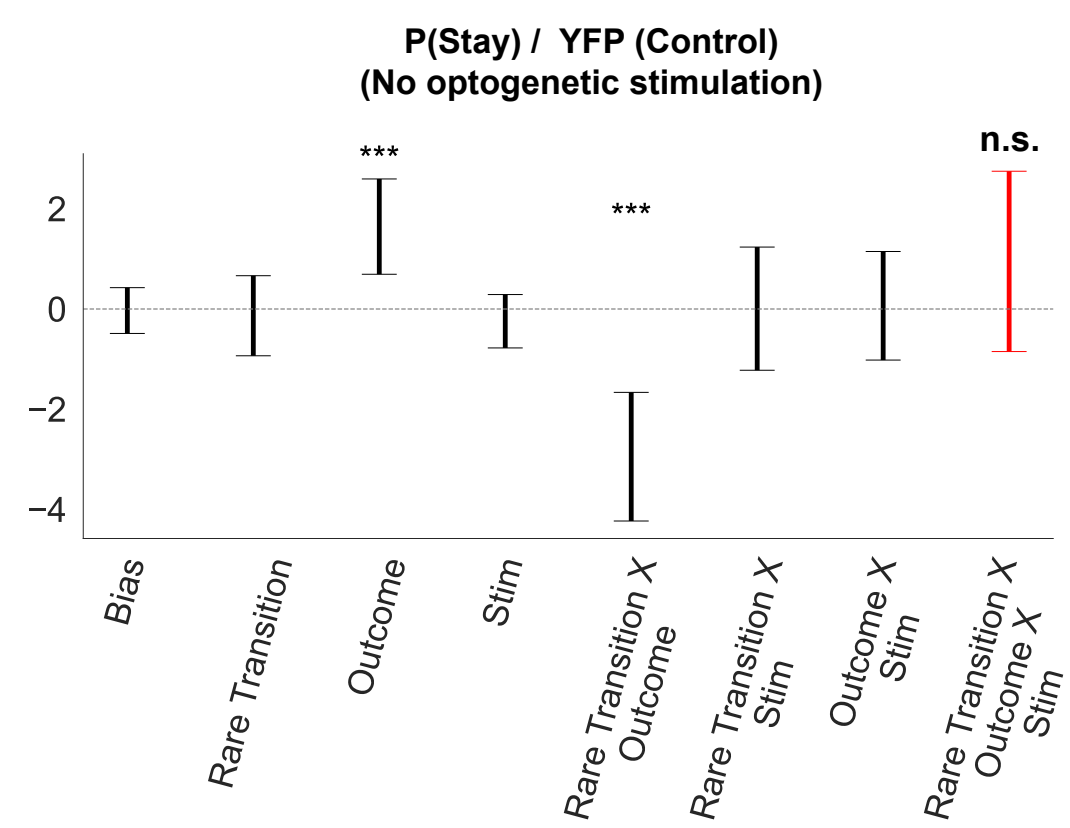

Figure S7: Regression analysis of dopamine stimulation effects in control animals. Unlike the ChR2 group, the three-way interaction (red), associated with enhanced transition learning from stimulation, did not differ significantly from chance levels in control animals ($p = 0.3$). Error bars show ±2 SE (approx. 95 % CI).

bioRxiv preprint doi: https://doi.org/10.64898/2026.02.18.706628; this version posted March 5, 2026. The copyright holder for this preprint (which was not certified by peer review) is the author/funder, who has granted bioRxiv a license to display the preprint in perpetuity. It is made available under a CC-BY 4.0 International license.

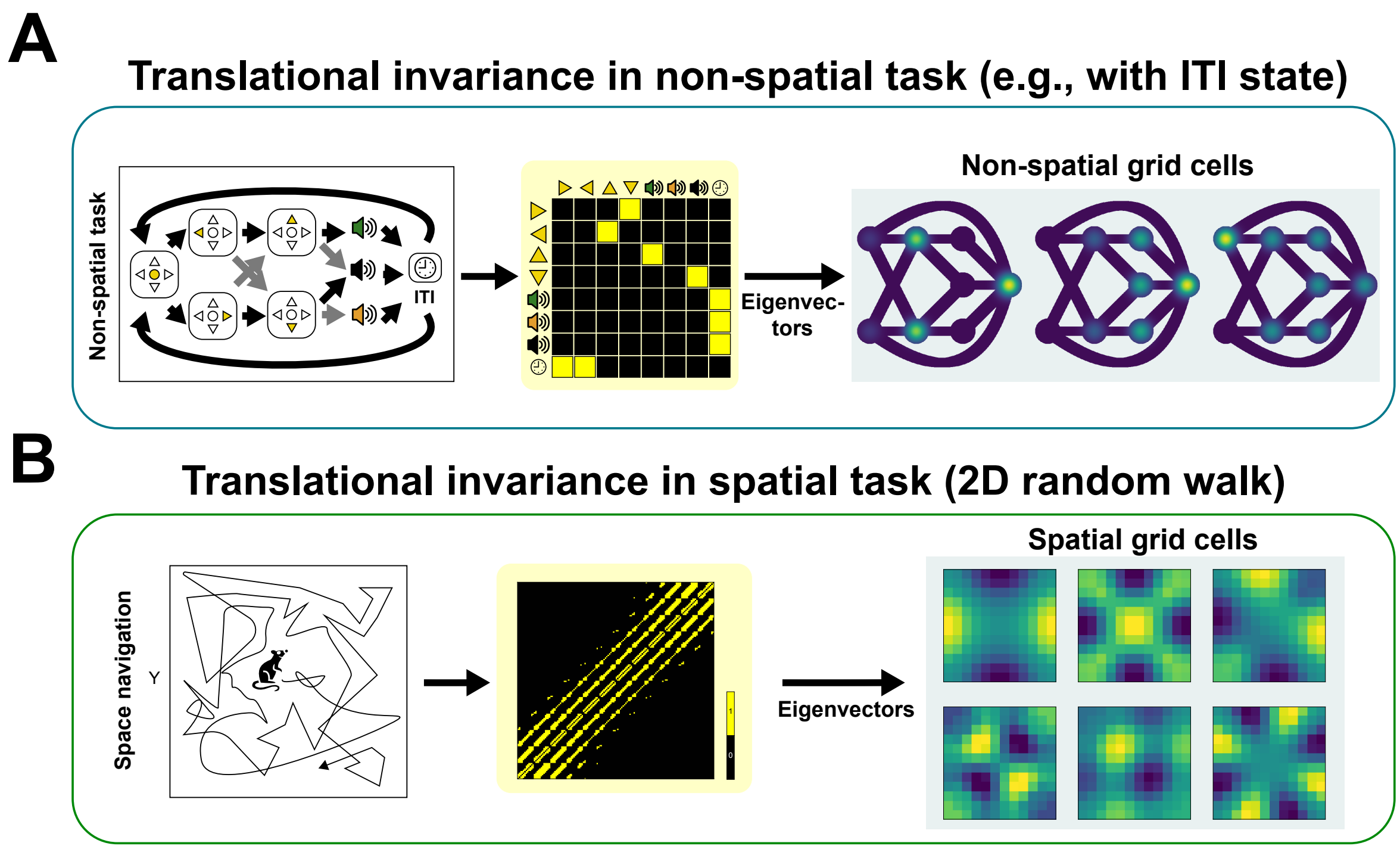

Figure S8: Grid-like and flag-like neural codes predicted by the SCG. (**A**) Grid-like codes in non-spatial tasks. Grid-like patterns emerge in trial-based decision tasks with temporal translational invariance. When the SCG forms cyclic graph structure, for example by including the inter-trial interval, it predicts grid-like activity over an abstract task space. (**B**) Grid-like codes in spatial tasks can also appear from the SCG with a relatively low threshold value.
